# Insights into the mechanism of SARS-CoV-2 main protease autocatalytic maturation from model precursors

**DOI:** 10.1038/s42003-023-05469-8

**Published:** 2023-11-13

**Authors:** Annie Aniana, Nashaat T. Nashed, Rodolfo Ghirlando, Leighton Coates, Daniel W. Kneller, Andrey Kovalevsky, John M. Louis

**Affiliations:** 1 Laboratory of Chemical Physics, National Institute of Diabetes and Digestive and Kidney Diseases, National Institutes of Health, DHHS, Bethesda, MD 20892-0520 USA; 2 Laboratory of Molecular Biology, National Institute of Diabetes and Digestive and Kidney Diseases, National Institutes of Health, DHHS, Bethesda, MD 20892-0520 USA; 3 Second Target Station, Oak Ridge National Laboratory, 1 Bethel Valley Road, Oak Ridge, TN 37831 USA; 4 Neutron Scattering Division, Oak Ridge National Laboratory, 1 Bethel Valley Road, Oak Ridge, TN 37831 USA; 5 Present Address: New England Biolabs, 240 County Road, Ipswich, MA 01938-2723 USA

**Keywords:** Proteases, X-ray crystallography, Proteolysis

## Abstract

A critical step for SARS-CoV-2 assembly and maturation involves the autoactivation of the main protease (MPro^WT^) from precursor polyproteins. Upon expression, a model precursor of MPro^WT^ mediates its own release at its termini rapidly to yield a mature dimer. A construct with an E290A mutation within MPro exhibits time dependent autoprocessing of the accumulated precursor at the N-terminal nsp4/nsp5 site followed by the C-terminal nsp5/nsp6 cleavage. In contrast, a precursor containing E290A and R298A mutations (MPro^M^) displays cleavage only at the nsp4/nsp5 site to yield an intermediate monomeric product, which is cleaved at the nsp5/nsp6 site only by MPro^WT^. MPro^M^ and the catalytic domain (MPro^1-199^) fused to the truncated nsp4 region also show time-dependent conversion in vitro to produce MPro^M^ and MPro^1-199^, respectively. The reactions follow first-order kinetics indicating that the nsp4/nsp5 cleavage occurs *via* an intramolecular mechanism. These results support a mechanism involving an N-terminal intramolecular cleavage leading to an increase in the dimer population and followed by an intermolecular cleavage at the C-terminus. Thus, targeting the predominantly monomeric MPro precursor for inhibition may lead to the identification of potent drugs for treatment.

## Abstract (web summary)

Investigation of the autoproccessing mechanism of SARS-CoV-2 main protease precursors reveals an N-terminal intramolecular cleavage event that leads to increased dimer formation and is followed by an intermolecular cleavage step at the C-terminus.

## Introduction

Specific cleavages of the polyproteins catalyzed by the virally encoded protease to release the mature structural and functional proteins are indispensable for viral assembly and maturation^1–3^. A single copy of the main protease of SARS-CoV2 (nsp5 or MPro) is translated from overlapping reading frames in two large polyproteins (pp) 1a and 1ab that encompass all non-structural proteins (nsps)^4–6^. A critical step in the regulation of MPro is the formation of a fully active mature MPro *via* autoprocessing at its termini^2,7^. MPro of SARS-CoV and SARS-CoV-2 are 96% identical in sequence and thus are similar in structure and function^8^. Both enzymes are ~68 kDa homodimers with each protomer having one active site C145-H41 catalytic dyad^7,9^. The protomer is composed of 306 amino acids and organized in three domains, I-III. Domains I (residues 8–101) and II (102–184) exhibit a chymotrypsin-like fold and connected to the helical domain III (residues 201–306) through a long loop region (residues 185–200)^9,10^. Structural studies indicate that monomeric forms are similar in their tertiary fold to the wild-type dimer except for differences in the active site oxyanion loop, the N-finger region and its interface, and domain III orientation^7,8,11^. Upon dimerization, the oxyanion loop (residues 137–144) assumes an active wound conformation (E*), whereas it is predominantly in an inactive unwound conformation (E) in the monomeric form^7,11,12^. Dimer interface mutants that are monomeric or predominantly monomeric bind to known inhibitors of wild-type MPro accompanied by the reorganization of the oxyanion loop to the active E* conformation typical of the dimer^11,13^. Also, inhibitor or substrate favor binding to the dimeric form of the enzyme^13,14^.

The spatio-temporal regulation of MPro release from its polyprotein precursor in vivo is predicted to be complex because of the association of its flanking nsp4 and nsp6 proteins with membranes^6,15–17^. Studies aimed at understanding the early mechanisms of maturation of the viral proteases from polyproteins are also complicated by the fact that polyproteins contain multiple cleavage sites and undergo autoprocessing during expression and purification^6^. In addition, our attempts to refold MPro from chemical denaturants were unsuccessful for in vitro studies. In this study, we have engineered several model precursors containing mutations that restrict dimer formation and thus facilitate the accumulation and purification of intact precursors from *E. coli*. These precursors undergo time-dependent autoprocessing reaction mediating cleavages at the termini of MPro in *E. coli* and in vitro and thus, provide simple models to study the mechanisms of MPro autocatalytic maturation.

## Results

### Order of cleavage at the termini of MPro from model precursors in *E. coli*

**Fig. 1:**
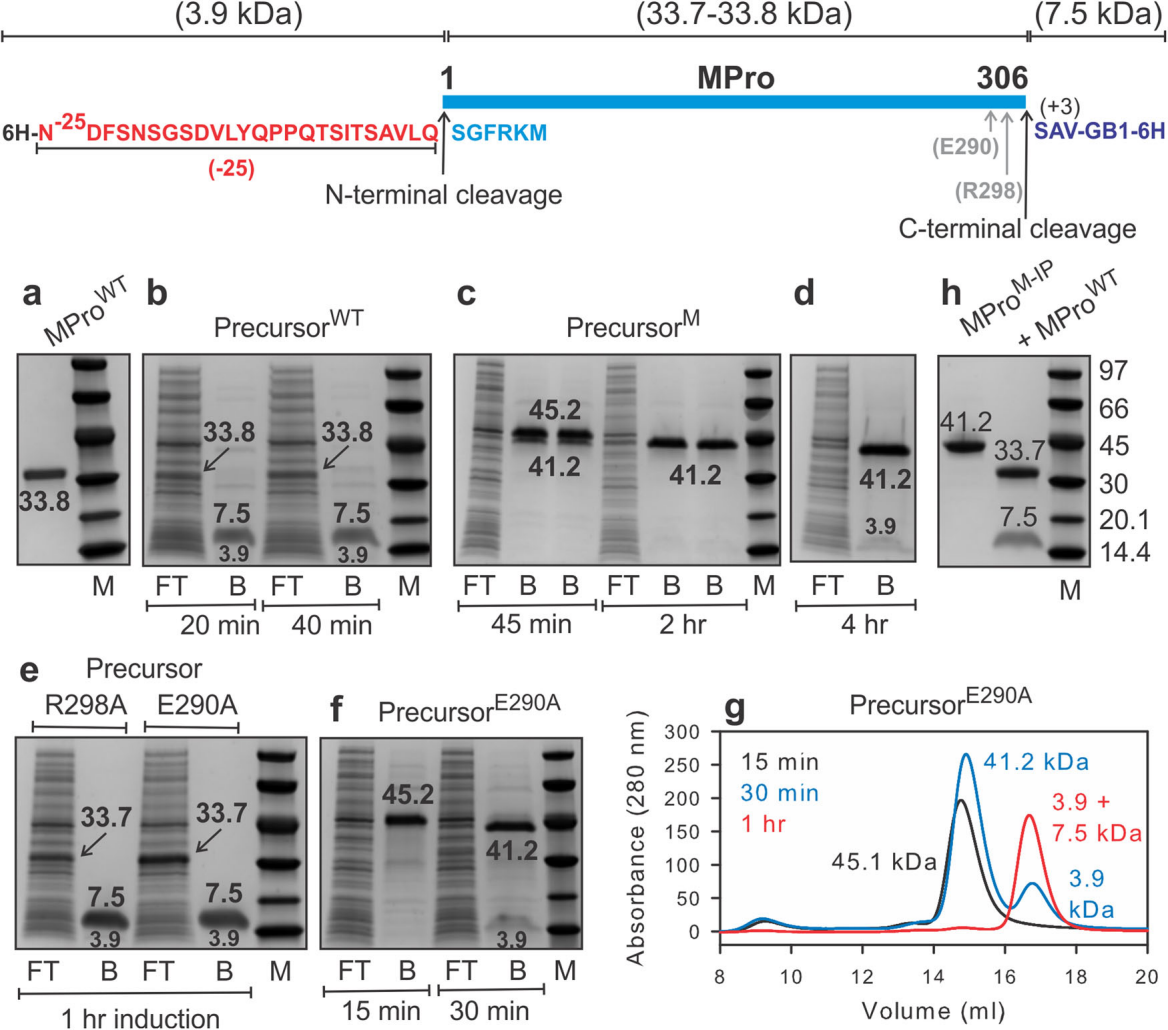
Autoprocessing of precursor mimetics of MPro containing native cleavage sites at both termini in *E.coli*. The MPro model precursor comprising the nsp4/nsp5 and nsp5/nsp6 cleavage sites is shown on top. **a**–**f** 12 ml of cells were harvested at the time points indicated below the lanes, subjected to NAC and equal amounts (3–4 µg) of the flow-through (FT) and bound (B) fractions were analyzed by SDS-PAGE. Molecular weight standards (M) are indicated in kDa. **a** Migration of purified mature MPro^WT^ (33.8 kDa). **b** Autoprocessing of precursor^WT^ results in 33.8 kDa mature MPro^WT^ in the FT, and 3.9 kDa and 7.5 kDa products in the B fractions. Cleavage products were verified by mass spectrometry. **c** Autoprocessing of precursor^M^ (45.2 kDa) results in the appearance of an intermediate product (41.2 kDa, MPro^M-IP^) upon cleavage at the N-terminus of MPro^M^, seen as a doublet together with the full-length protein at 45 min of induction, and only that of MPro^M-IP^ at 2 hr of induction in the bound B fractions. **d** No C-terminal cleavage occurs even after 4 h of induction as indicated by the absence of the mature MPro^M^ (33.7 kDa) in the FT. **e, f** Autoprocessing of Precursor^R298A^ and Precursor^E290A^. **g** Fractionation of the bound fractions of Precursor^E290A^ at 15, 30 (**f**) and 60 min (**e**) on Superose 12 (1 × 30 cm column) in buffer A confirming the stepwise cleavage at the N-terminus first, followed by the cleavage at the C-terminus as seen on gels. The precursor and products of cleavage were verified by mass spectrometry. **h** Cleavage of the nsp5/nsp6 site of MPro^M-IP^ by MPro^WT^.

Our simplified strategy entails expressing MPro (nsp5) containing flanking region sequences of nsp4 and nsp6 as a precursor mimetic. Recent studies indicate that the addition of non-native residues to the N-terminus of MPro results in a drastic decrease in catalytic activity as compared to such residues appended to the C-terminus of MPro^18^. The decrease in catalytic activity is consistent with our earlier observation that the addition of six native nsp4 residues (TSAVLQ) to the N-terminus of MPro increases the dimer dissociation constant^12^. These results imply that the reorganization of the free N-terminal residues of MPro upon N-terminal autoprocessing (nsp4/nsp5 site) through specific key interactions with domain III leads to enhanced dimer stability and appearance of mature-like catalytic activity, similar to that previously described for SARS-CoV MPro^7,19^. Thus, our design strategy for MPro precursor contained 25 C-terminal residues of nsp4 appended to the N-terminus of MPro [^(−25)^MPro] to enable discerning the migration of the precursor from the mature MPro upon autoprocessing by SDS-PAGE without compromising solubility. Likewise, extending the length of the flanking nsp4 even up to 102 residues ^(−102)^MPro resulted in the production of soluble protein and with a K_dimer_ that is predicted to be like that of ^(−25)^MPro^11,13^. Constructs and their designations used in this study are shown in the corresponding main figures and listed in Figure S1. Since appending even 25 residues of nsp6 to MPro causes severe insolubility of the expressed protein, the soluble immunoglobulin binding domain B1 of Protein G [GB1^20^,], preceded by 3 N-terminal residues of nsp6 (SAV) to retain nsp5/nsp6 cleavage site specificity, were used (Fig. 1). This strategy allows monitoring native cleavages at both termini of MPro as a fully soluble precursor in *E. coli* and in vitro. Expression of Precursor^WT^ results in rapid autoprocessing at both sites within 20 min and no full-length precursor or intermediate product is observed (Fig. 1b). The products corresponding to mature MPro^WT^ (33.8 kDa, Fig. 1a) and the terminal fragments were identified (Fig. 1b), and the purified terminal fragments were confirmed by mass spectrometry.

**Fig. 2:**
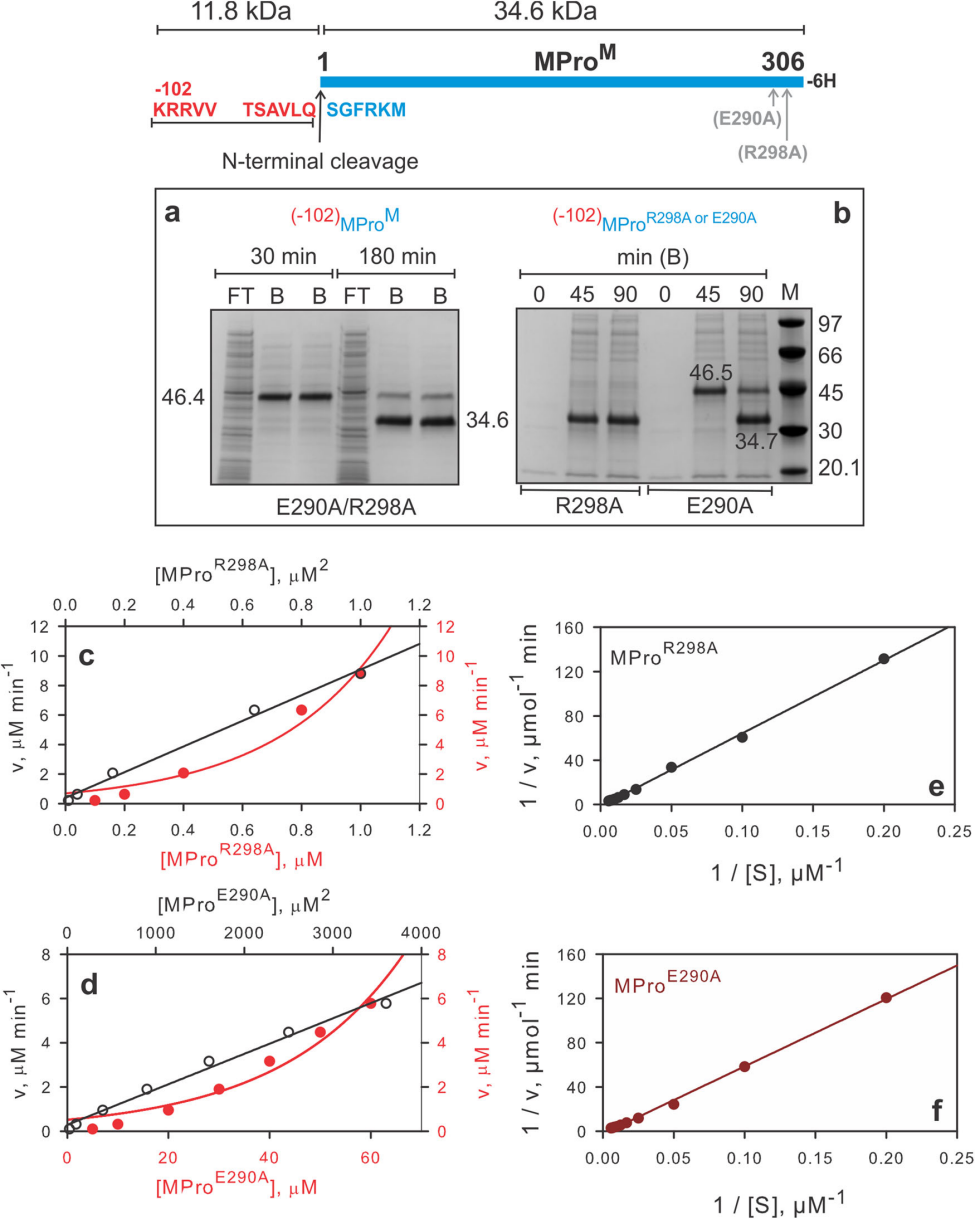
N-terminal autoprocessing of MPro^M^ precursor and its single mutants in *E. coli* and characterization of products. The N-terminal cleavage site is indicated with an upward black arrow. Precursor, products released upon cleavage at the N-terminus of MPro and molecular weight standards (M) are indicated in kDa. The gels show the time course of the autoprocessing reaction of MPro^M^ (**a**) and single mutants R298A and E290A **(b)** with 102 amino acids of the nsp4 sequence appended to the N-terminus of MPro. Cells (12 ml) were harvested at the time points indicated, subjected to NAC, and analyzed by SDS-PAGE. FT and B denote flow-through and bound fractions, respectively, after NAC. The 11.8 kDa product is not observed in the bound fraction because it lacks the 6His-tag. **c**–**f** Catalytic activity of mature MPro^R298A^ and MPro^E290A^. **c, d** Linear relationship between the rate *vs* the square of the protein concentration assayed with 200 µM substrate. **e, f** Lineweaver-Burk plots for the hydrolysis of substrate by 0.2 μM MPro^R298A^ and 3.5 μM MPro^E290A^ in the presence of equimolar concentration of GC373. These concentrations represent the K_d_ estimated by ITC like that described for MPro^M^ ^13^. The k_cat_/K_m_ was calculated as described^13^, and compared with previously characterized constructs shown in Table 2^11,13^.

Since the catalytic activity inversely correlates with the dimer dissociation constant [K_dimer_^11,21^,], it is predictable that mutations of residues contributing to dimer formation would enable the production of a functional precursor for monitoring the autoprocessing reaction. Thus, interface residues E290 and R298 were targeted for mutations in Precursor^WT^. In earlier studies, we had shown that these mutations together increase the K_dimer_ of mature MPro by 5000-fold^13^ and accordingly this mutant was termed MPro^M^ (where ^M^ denotes monomer). Precursor^R298A^ undergoes rapid autoprocessing at both termini, like that of Precursor^WT^, i.e., complete conversion within 1 h of expression to release the mature MPro^R298A^, 3.9 kDa and 7.5 kDa products (Figs. 1e, 2b). In contrast, Precursor^E290A^ processing is observed to be much slower than Precursor^WT^ and Precursor^R298A^ allowing the transient observation of the time-dependent stepwise autoprocessing at both termini (see Fig. 1f). The expressed protein being fully soluble enabled the purification of the full-length protein and its product by nickel-affinity chromatography (NAC) expeditiously under native conditions. At 15 min, only Precursor^E290A^ (45.1 kDa) is detectable in the bound (B) fraction, followed by the appearance of the intermediate species of 41.2 kDa and the 3.9 kDa corresponding to the products of the N-terminal cleavage at 30 min. The 41.2 kDa product is then converted to the mature MPro^E290A^ and the 7.5 kDa product upon C-terminal cleavage at 1 h of induction (Fig. 1e). This is further confirmed by analyses of the bound fractions at 15, 30 and 60 min by size-exclusion chromatography (Fig. 1g) and mass spectrometry.

The double mutant E290A and R298A precursor (Precursor^M^) also exhibits its accumulation in *E. coli* and time-dependent autoprocessing reaction, but at a slower rate than that of Precursor^E290A^. Analysis of the fractions indicates cleavage at the N-terminus of MPro^M^ leading to the accumulation of the intermediate product (MPro^M-IP^, 41.2 kDa, Fig. 1c) after 2 h of expression with no detectable product indicative of a C-terminal cleavage even after 4 h (Fig. 1d). The identity of MPro^M-IP^ was verified by mass spectrometry (41,180 Da, calculated = 41,180 Da). Nevertheless, incubation of MPro^M-IP^ with MPro^WT^ at a ratio of 50:1 produced the previously characterized mature MPro^M^ ^13^ indicating that the C-terminal cleavage site is accessible for reaction (Fig. 1h).

### N-terminal autoprocessing of MPro precursor analogues in *E.coli*

Like Precursor^WT^, expression of MPro^WT^ with only the native nsp4/nsp5 cleavage site also results in the lack of accumulation of the precursor, with autoprocessing complete within 15 min of expression to release mature MPro^WT^ [Fig. S2a^11^,]. An active site C145A mutation abolishes this cleavage (Fig. S2a^11^,).

We had previously reported the expression of a construct consisting of 25 amino acids of the nsp4 sequence appended to the N-terminus of the double mutant MPro^M^ as a miniprecursor, ^(−25)^MPro^M^, which undergoes time-dependent autoprocessing upon expression in *E. coli* [Fig. S2b^11^,]. The 37.4 kDa precursor is converted to the 34.6 kDa and 2.8 kDa products at a much slower rate relative to the MPro^WT^ precursor. Only the 34.6 kDa product (MPro^M^) containing the 6H-tag allows its detection together with the precursor after NAC. To better resolve the separation between the starting material and products of the autoprocessing reaction by SDS-PAGE, a similar set of constructs comprising 102 amino acids of nsp4 appended to the N-terminus of MPro^WT^, MPro^E290A^, MPro^R298A^, and MPro^M^ were expressed in *E. coli* (Fig. 2). Like Precursor^WT^ and its mutants, expression of ^(−102)^MPro^WT^ and ^(−102)^MPro^R298A^ (Fig. 2b) resulted in observing only the processed products released upon N-terminal autoprocessing. Time course of the autoprocessing of ^(−102)^MPro^WT^ parallels that of the wild-type precursor shown in Figure S2A. Precursor ^(−102)^MPro^E290A^ and ^(−102)^MPro^M^ exhibit transient accumulation of the full-length precursor followed by N-terminal cleavage to yield products (Fig. 2a, b) like that of Precursor^E290A^ and Precursor^M^ (and ^(−25)^MPro^M^, Fig. S2c), respectively, undergoing N-terminal cleavage.

### N-terminal autoprocessing of MPro precursor analogues in vitro

**Fig. 3:**
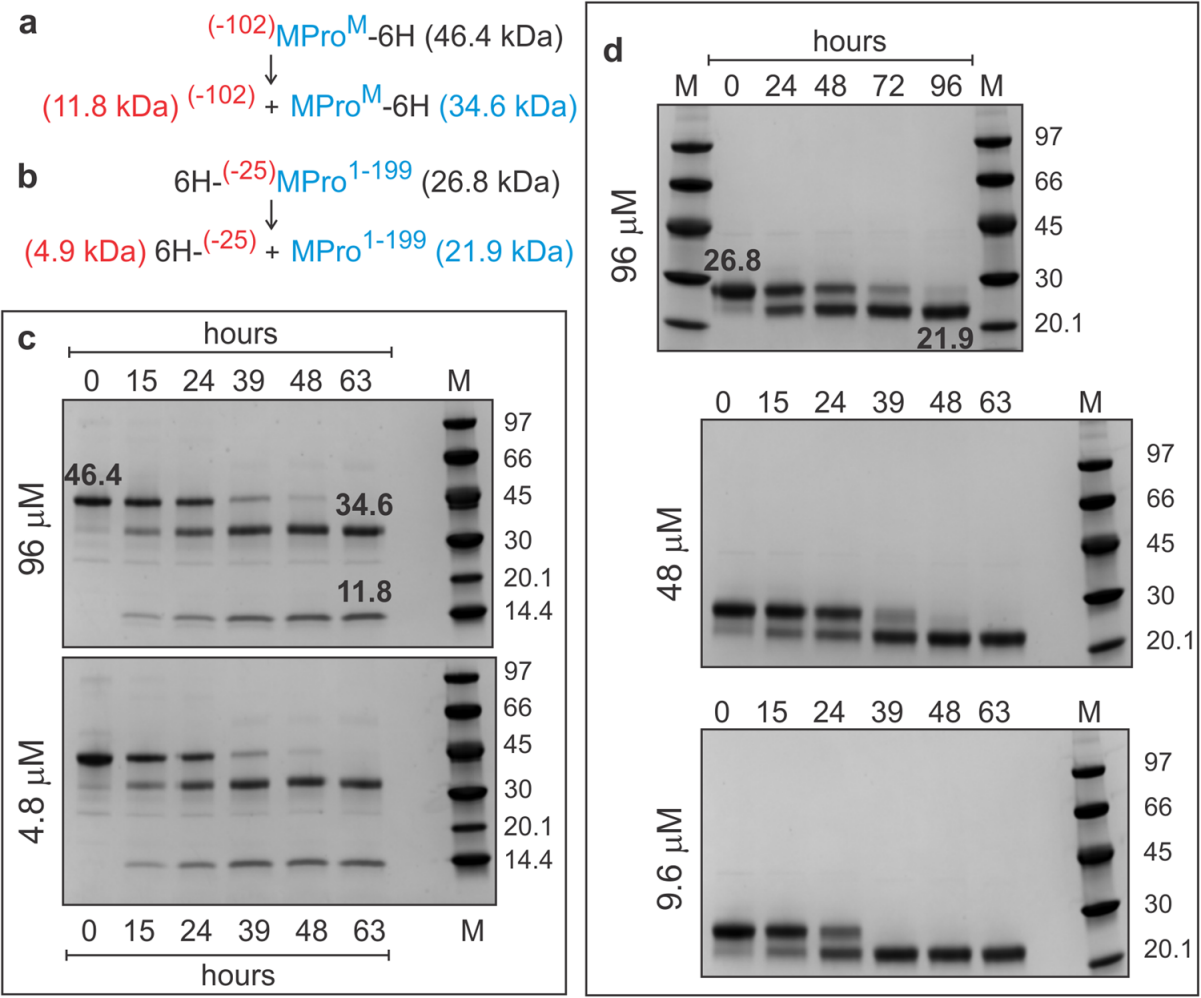
Time course of the autoprocessing of ^(−102)^MPro^M^ and ^(−25)^MPro^1–199^ as a function of decreasing protein concentration. **a, b** Precursor constructs showing the products released upon N-terminal autoprocessing. Time course of (**c**) ^(−102)^MPro^M^ and (**d**) ^(−25)^MPro^1–199^ autoprocessing. Reactions were initiated at the indicated concentration in buffer B at 28 °C (see Tables S1, S2 for details). Aliquots of the reaction were drawn at the indicated times and terminated by the addition of gel sample buffer. Samples (2.6–3.6 µg/lane) were subjected to SDS-PAGE and visualized by staining. Precursor, reaction products, and molecular weight standards (M) are indicated in kDa.

Interestingly, like ^(−25)^MPro^M^, precursor ^(−25)^MPro^1–199^ lacking the entire helical domain (residues 200–306), as a monomer, also undergoes time-dependent N-terminal autoprocessing upon expression in *E. coli* to produce MPro^1–199^ [Fig. S2c^11^,]. Transient accumulation of full-length precursors ^(−102)^MPro^M^, ^(−25)^MPro^M^ and ^(−25)^MPro^1–199^ allowed their purification in a small scale to yield sufficient protein for in vitro analyses. Details of the reaction set-up are shown in Tables S1, S2. Samples collected at various time points were subjected to SDS-PAGE. Figure 3 shows the time course of the reactions. For ^(−102)^MPro^M^ and ^(−25)^MPro^199^, the starting material and the products are well separated on gels allowing quantifying the band intensities. The percent conversion at 15 and 24 h of incubation are calculated from each initial protein concentration and shown in Table 1. In addition, precursor ^(−102)^MPro^M^-catalyzed hydrolysis of the FRET substrate was monitored. The intrinsic catalytic activity was estimated to be 0.0204 μM/h when assayed with 50 μM precursor and 200 μM substrate. After incubation of ^(−102)^MPro^M^ for 24 h, the observed value is 0.0384 μM/h, pointing to a 75% increase in catalytic activity attributable to the N-terminal cleavage of ^(−102)^MPro^M^.

**Table 1:** Time-dependent N-terminal autoprocessing of MPro in vitro.

Construct	[P_o_] µM	At 15 h		At 24 h	
		Δ[P], µM	% Conversion	Δ[P], µM	% Conversion
^(−102)^MPro^M^	96	36.9 ± 2	38.4	53.7 ± 3	55.9
	4.8	1.93 ± 0.1	40.2	2.8 ± 0.2	59.2
^(−25)^MPro^1–199^	96			40.5 ± 0.4	42.2
	48	15.4 ± 0.2	32.2	20.7 ± 0.2	43.2
	9.6	3.1 ± 0.1	32.4		

Band intensities shown in Fig. 3 were quantified using the ImageJ software (https://imagej.nih.gov/ij/). [P_0_] denotes the precursor ^(−102)^MPro^M^ (46.4 kDa) and ^(−25)^MPro^1–199^ (26.8 kDa) concentrations at time zero and Δ[P] is the amount of [P_0_] converted to products upon incubation for 15 and 24 h, calculated from the sum of the band intensities at each time point.

**Fig. 4:**
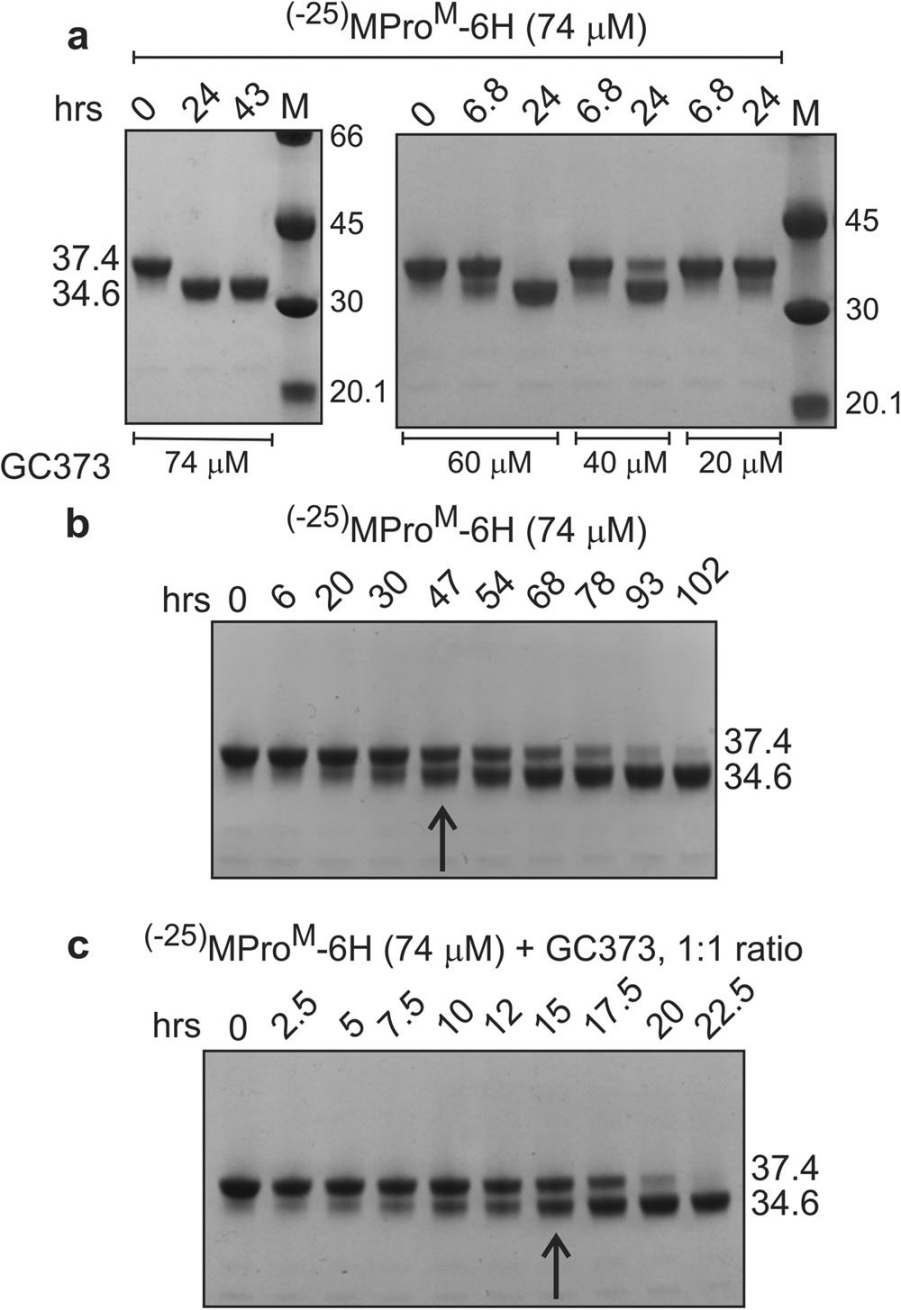
Time course of the autoprocessing of ^(−25)^MPro^M^ in the absence and presence of GC373. **a** Autoprocessing monitored by varying the inhibitor GC373 concentration. Time course of autoprocessing in the absence (**b**) and presence (**c**) of GC373. Reactions were initiated at the indicated concentration in buffer B at 28 °C. Aliquots of the reaction were drawn at the indicated times, terminated by the addition of gel sample buffer, subjected (3.5 µg/lane) to SDS-PAGE and visualized by staining. Precursor, reaction product, and molecular weight standards (M) are indicated in kDa.

Like MPro^M^ ^13^, the autoprocessing of ^(−25)^MPro^M^ precursor is accelerated in a concentration-dependent manner by GC373 up to an equimolar concentration (Fig. 4a). Figure 4b, c show qualitatively that, similar amount of MPro^M^ is produced from ^(−25)^MPro^M^ in 15 h in the presence of equimolar amount of GC373 as compared to that in 47 h without GC373.

### Characterization of products of the autoprocessing reactions

**Fig. 5:**
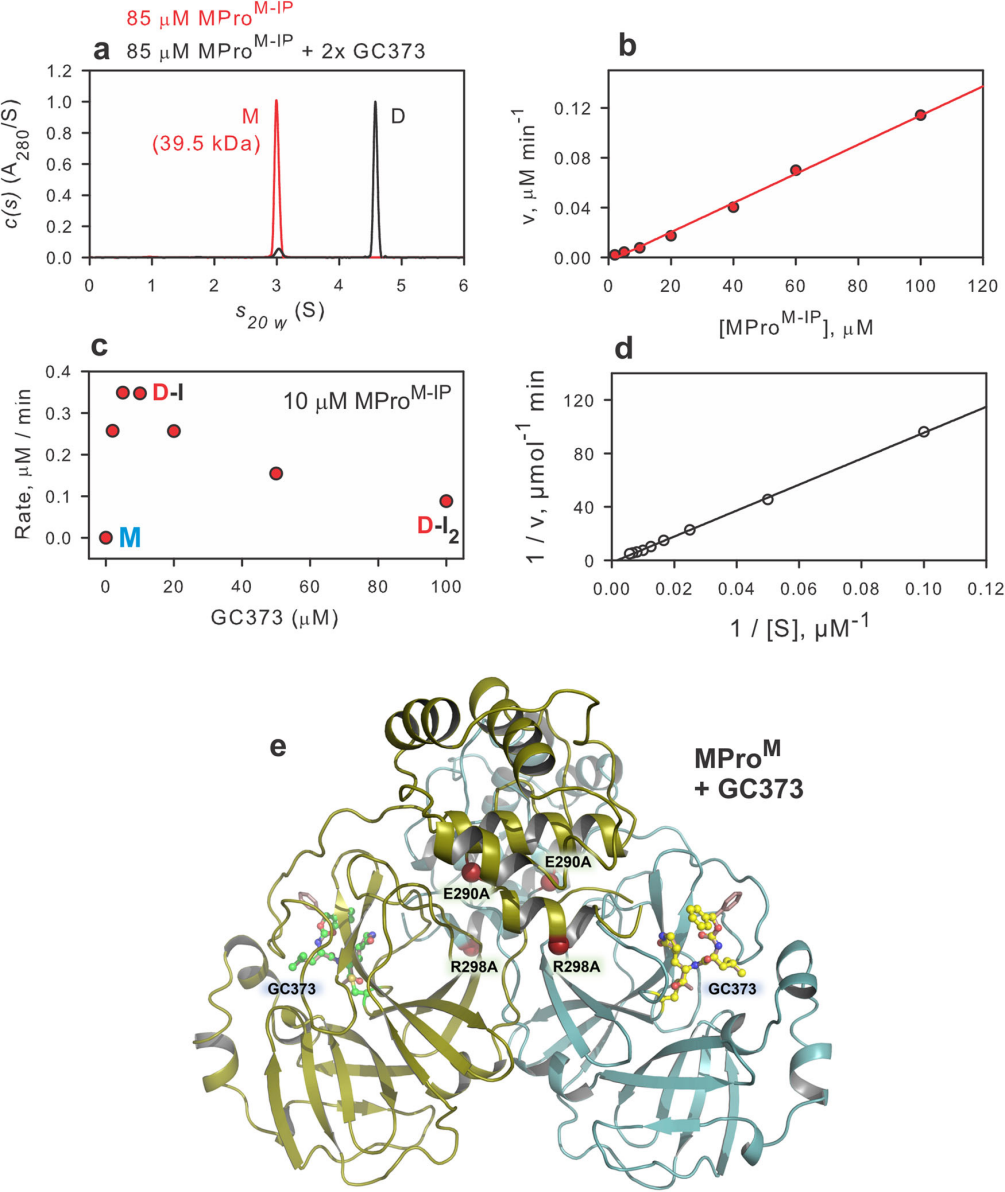
Characterization of MPro^M-IP^ and ribbon representation of room temperature structure of GC373 bound MPro^M^ dimer. **a** Normalized sedimentation velocity absorbance c(s) distributions in the absence (red) and presence (black) of GC373. **b** Linear relationship between the rate of catalyzed hydrolysis *vs* concentration. **c** A plot of the rate as a function of increasing GC373 mixed with a constant amount of MPro^M-IP^. **d** Lineweaver-Burk plot for the hydrolysis of substrate by 10 μM MPro^M-IP^ in the presence of equimolar concentration of GC373. The k_cat_/K_m_ was calculated as described^13^ and listed in Table 2. **e** Room temperature structure of GC373 bound MPro^M^ (PDB 8FIG). The positions of mutations E290A and R298A used to create MPro^M^ with a very high K_dimer_^13^ and GC373 are shown.

The intermediate product MPro^M-IP^ resulting from the N-terminal autoprocessing of Precursor^M^, was purified from cells expressed for 2.5 h as described in Methods. It is monomeric in solution as shown by SV-AUC up to a concentration of 85 μM (Fig. 5a). It catalyzes the hydrolysis of the FRET substrate, and the rate of hydrolysis of the peptide substrate displays first-order kinetics in protein concentration, i.e., a linear dependency on the protein concentration at a constant substrate concentration (Fig. 5b). This further confirmed that the observed catalytic activity is that of a monomer, and no detectable amount of dimer is present in solution. Unlike mature MPro^M^, the kinetics of MPro^M-IP^ catalyzed hydrolysis is similar to that of monomeric MPro^1–199^ [^11^ and Table 2)]. However, like MPro^M^ ^13^, MPro^M-IP^ binds to GC373 with concomitant dimer formation and enhanced catalytic activity up to an equimolar concentration of the MPro^M-IP^ and GC373 (Fig. 5c). Above the equimolar amount, the catalytic activity is inhibited by increasing the concentration of GC373. The kinetic parameter k_cat_/K_m_ for MPro^M-IP^ in the absence and presence of GC373 as well as the binding constant to GC373 determined by SV-AUC and ITC are listed in Table 2 (Fig. 5d, S3, S4) along with those of MPro^WT^, MPro^M^ and MPro^1–199^. Although the 7.5 kDa SAV-GB1-6H fragment corresponding to the product of the C-terminal nsp5/nsp6 cleavage of MPro^M-IP^ by MPro^WT^ is easily observed (Fig. 1h), surprisingly no cleavage is detected upon incubation of MPro^M-IP^ in the absence or presence of equimolar amount of GC373 up to 48 h.

**Table 2:** Catalytic activity, dimer dissociation and GC373 binding constants of mature MPro and its precursor analogues.

Construct	k_cat_/K_m_ (µM^−1^ min^−1^)	k_cat_/K_m_K_dimer_^a^ (µM^−2^ min^−1^)	K_dimer_ (µM)	GC373 binding (K_d_, µM)	Catalytic/Binding species
**Wild-type**					
MPro^WT b^	0.6 ± 0.05		1.3 ± 0.2	0.15 ± 03 ^c^	Dimer
**Substitution mutants**					
MPro^R298A^	0.31 ± 0.01 ^d^	0.0435 ± 0.002	7.13 ± 0.40	0.2 ± 0.04 ^c^	Dimer
MPro^E290A^	(2.82 ± 0.04) 10^−3 d^	(8.0 ± 0.40) 10^−6^	353 ± 21	3.5 ± 0.5 ^c^ 4.8 ± 0.6 ^e^	Dimer
MPro^M b^	(3.4 ± 0.13) 10^−3^	(5.0 ± 0.15) 10^−7^	6600	6.13 ± 0.3 ^c^ 6.7 ± 0.2 ^e^	Dimer
**Deletion mutant**					
MPro^1–199 b^	(1.15 ± 0.03) 10^−6^			32 ± 5 ^c^	Monomer
**Precursor analogues**					
^−102^MPro^M^				19.2 ± 4.4 ^c^	Monomer
MPro^M-IP^	(6.04 ± 0.15) 10^−6^				Monomer
	(9.40 ± 0.90) 10^−4 d^			6.02 ± 1.2 ^c^ 8.5 ± 1.5 ^e^	Dimer

^a^k_cat_/K_m_K_dimer_ is the slope of the linear plot of the rate *vs* the square of the protein concentration.

^b^cited from references ^11,13^.

^c^determined by ITC.

^d^k_cat_/K_m_ was determined in the presence of the equimolar amount of enzyme and GC373 as described for MPro^M^ in Nashed et al.^13^.

^e^determined by SV-AUC.

The 6H-tag at the C-terminus facilitates the initial purification of the MPro products derived from the autoprocessing of ^(−102)^MPro^E290A^ and ^(−102)^MPro^R298A^ and was removed to produce mature MPro^E290A^ MPro^R298A^ as previously described for MPro^WT^ ^9,13^. MPro^R298A^ and MPro^E290A^ exhibit a dimer dissociation constant (K_dimer_) of 7.1 ± 0.4 µM and 353 ± 21 µM, respectively, compared to 6.6 mM for the double mutant MPro^M^ (Table 2 and Fig. S5). Purified mature MPro^E290A^ and MPro^R298A^ catalyze the hydrolysis of the peptide substrate, and the rate of hydrolysis is linearly dependent on the square of the protein concentration, i.e., second-order kinetics, indicating that the observed catalytic activity is that of a dimer like that of MPro^WT^ and MPro^M^ (Fig. 2c, d). The kinetic parameters k_cat_/K_m_ of mature MPro^E290A^ MPro^R298A^- catalyzed hydrolysis of substrate as well as the inhibitor GC373 binding constants are listed in Table 2 (Figs. 2e, f, S4a, b, S5c). The monomer-dimer distributions of MPro^R298A^ and MPro^E290A^ in the absence of inhibitor or substrate by SV-AUC are shown in Figure S5a, b.

### Room-temperature structure of GC373 bound MPro^M^

**Fig. 6:**
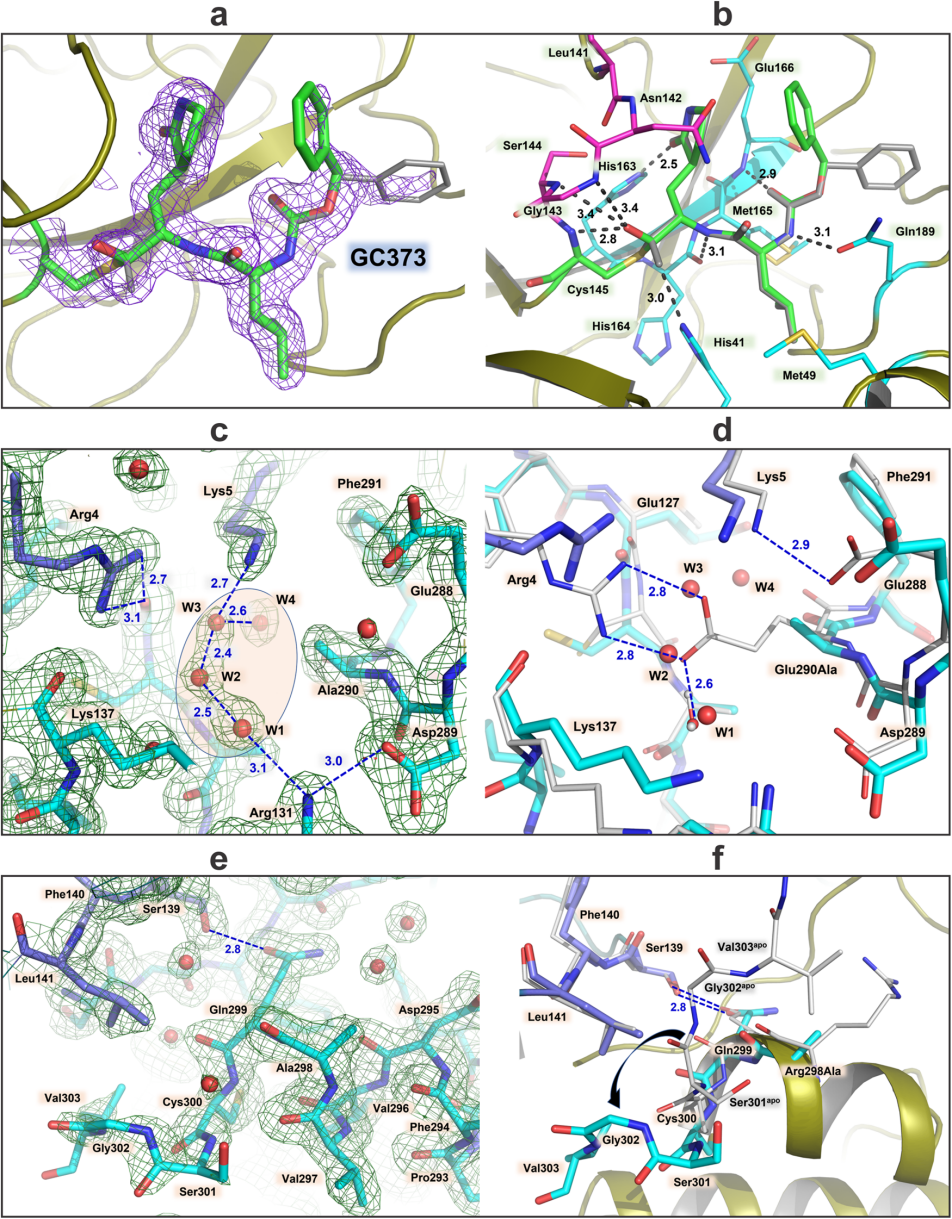
Binding of GC373 to MPro^M^ and the effects of mutations on the dimer interface. **a** 2F_O_-F_C_ electron density of GC373 covalently bonded to C145 is shown at 1.5 σ level. GC373 binds in two alternate conformations shown with green carbon atoms and in gray with the refined occupancies of 51/49%, respectively. **b** Hydrogen bonds formed by GC373 with the MPro^M^ active site residues. Active site and oxyanion hole residues are colored by atom type with cyan and magenta carbon atoms, respectively. **c** 2F_O_-F_C_ electron density for the interprotomer interface near E290A mutation site shown at 1.5 σ level. Protomer A carbon atoms are colored cyan, whereas protomer B carbon atoms are colored slate. **d** Superposition of MPro^M^ (cyan and slate carbon atoms) and MPro^WT^ (gray carbon atoms) near E290A mutation site demonstrating the loss of the E290…R4’ salt bridge. **e** 2F_O_-F_C_ electron density for the interprotomer interface near R298A mutation site is shown at 1.5 σ level. **f** Superposition of MPro^M^ and MPro^WT^ near R298A mutation site showing a conformational reorientation of the C-terminal residues (black curved arrow). Atoms are colored in the same way as in panel **d**. All distances are shown in Angstroms.

Since MPro^M^ is predominantly monomeric in solution, the effect of the mutation of residues E290A and R298A on the overall structure of the protein particularly in the active site region and the monomer-dimer interface regions that include the N- and C-termini is examined by X-ray crystallography. Attempts to grow crystals of monomeric MPro^M^ and MPro^M-IP^ were unsuccessful, although a crystal structure of the monomeric SARS-CoV-1 MPro^R298A^ single mutant was attained previously^22^. However, we succeeded in obtaining a room-temperature crystal structure of MPro^M^ in complex with the reversible covalent inhibitor GC373 at 1.75 Å resolution (Table 3). MPro^M^ crystallizes in the monoclinic unit cell (space group *I*2) with one protomer present in the asymmetric unit. MPro^M^-GC373 complex is homodimeric, with the quaternary structure virtually identical to that of the MPro^WT^ [Fig. 5e^10,23^]. We modeled residues 1-303 in MPro^M^-GC373 structure, whereas the rest of the C-terminal residues are disordered and are not visible in the electron density map. GC373 is covalently bonded to the sulfur of the catalytic Cys145 generating the hemithioacetal conjugate. Interestingly, the nucleophilic attack of the sulfur of Cys145 on the carbonyl carbon of GC373 is not stereospecific and occurs from both sides of the carbonyl carbon such that the hemithioacetal hydroxyl group is observed in two alternate orientations (Fig. 6a). In one orientation, the hydroxyl is directed into the oxyanion hole created by residues Leu141-Cys145, whereas in the other it faces the catalytic His41. In addition, the phenyl tail of GC373 occupies two alternate conformations. In the previous room temperature X-ray structure of MPro^WT^-GC373 complex^11^, the ligand was found in one orientation, bound in a stereospecific fashion with the hemithioacetal hydroxyl directed into the oxyanion hole. GC373 makes five direct hydrogen bonds with MPro^M^ residues (Fig. 6b). The shortest and possibly the strongest hydrogen bond of 2.5 Å is formed between the ligand’s P1 γ-lactam and imidazole of His163 and is known to be a critical interaction for inhibitors with the MPro active site^24–26^. The remaining four hydrogen bond distances are significantly longer, 2.8–3.4 Å. It should be noted that the lack of stereospecificity in the reaction of the sulfur of C145 with the carbonyl carbon of GC373 is not caused by the mutations because (1) the isomeric hemithioacetals have been previously reported in the cryo-structure of MPro^WT^-GC373 complex^27^ and (2) the room temperature structure of MPro^1–199^-GC373 complex shows only the S-configuration for the hemithioacetal carbon^11^.

**Table 3:** Crystallographic data collection and refinement statistics.

	MPro^M^-GC373 PDB ID 8FIG
Data collection:	X-ray (in-house)
Diffractometer	Rigaku HighFlux, DECTRIS Eiger R 4 M
Space group	*I*2
Wavelength (Å)	1.5406
**Cell dimensions:**	
*a*, *b*, *c* (Å)	52.24, 80.76, 92.52
α, β, γ (°)	90, 95.6, 90
Resolution (Å)	60.71–1.75 (1.81–1.75)
No. reflections unique	36987 (3583)
*R*_merge_	0.065 (0.635)
*CC*_*1/2*_	0.996 (0.580)
*<I* / σ*I* >	18.7 (1.5)
Completeness (%)	95.9 (92.8)
Redundancy	5.5 (4.7)
**Refinement:**	
*R*_work_ / *R*_free_	0.1647 / 0.1847
Resolution (Å) ***B*-factors (Å**^**2**^**)**	60.71–1.75
Protein	37.16
Ligand	31.2
Water	43.34
**R.M.S. deviations**	
Bond lengths (Å)	0.005
Bond angles (°)	0.804
All atom clash score	1.26

Data reduction and refinement statistics for the room temperature X-ray crystal structure of SARS-CoV-2 MPro^M^-GC373 complex. Values in parentheses are for the highest-resolution shell.

The electron density map for the E290A mutation site located at the dimer interface is shown in Fig. 6c where interactions with the N-terminal finger (residues 1–6) occur. Four water molecules (W1-W4) are clearly visible in the vicinity of Ala290, making a tight hydrogen-bonded network with distances between the waters of 2.4–2.6 Å. The water structure intervenes between the stretch of residues 288–291 of protomer A and Arg4 and Lys5 of protomer B. W1 hydrogen bonds with Arg131 (protomer A), and W3 makes a hydrogen bond with Lys5 (protomer B). Thus, the waters mediate the connection between the two protomers. Arg4 of protomer B forms hydrogen bonds with the main chain carbonyl of Glu127 of protomer A. Evidently, there are no direct hydrogen bonds between the protomers in this region. When MPro^M^-GC373 is superimposed with the room-temperature joint X-ray/neutron structure of MPro^WT^ [PDB ID 7JUN^23^,] or with the room-temperature X-ray structure of MPro^WT^-GC373 complex [PDB ID 7UKK^11^,], several important structural differences relative to MPro^M^ can be noted (Figs. 6d and S6). In both MPro^WT^ structures, Glu290 of protomer A makes strong 2.6–2.8 Å hydrogen bond interactions with Arg4 of protomer B through a salt bridge structure. In addition, Glu288 of protomer A forms 2.9–3.0 Å hydrogen bonds with Lys5 of protomer B in the inhibitor-free and GC373-bound MPro^WT^ structures. In MPro^M^-GC373, these hydrogen bonds are severed due to shifts of Glu288 of protomer A and Lys5 of protomer B away from each other, resulting in their separation of 5.5 Å. Moreover, to stabilize its position in the absence of the Glu290 carboxylic group, the Arg4 of the protomer B side chain flips towards the main chain of Glu127. W1 is conserved between inhibitor-free MPro^WT^ and MPro^M^-GC373 structures and is replaced with the alternate conformation of Glu290 in MPro^WT^-GC373 complex. W1 makes a 2.6 Å hydrogen bond with Glu290 in MPro^WT^. Thus, because of the E290A mutation, three water molecules W2, W3, and W4 invade the dimer interface disrupting several hydrogen bonds between the protomers and contributing to the increase in the K_dimer_.

The electron density map for the MPro^M^ residues around the R298A mutation site located where C-terminal residues interact with the other protomer and specifically with Ser139-Leu141 turn at the start of the oxyanion loop is shown in Fig. 6e. There is one direct interprotomer hydrogen bond of 2.8 and 2.7 Å from Gln299 of protomer A to Ser139 of protomer B in inhibitor-free MPro^WT^ and MPro^WT^-GC373, respectively. This hydrogen bond is retained in MPro^M^-GC373 compared to MPro^WT^ when the structures are superimposed. In both MPro^WT^ structures, Arg298 is situated more than 5 Å from the nearest residue of the other protomer. However, it makes hydrophobic interactions with the C-terminal residues from the same protomer, which are lost when this Arg is mutated to Ala. In both MPro^WT^, the C-terminal residues starting from Cys300 located at the end of a short α-helix of residues 293–300 make a 90° turn towards the interface between the catalytic domains. In MPro^M^-GC373 instead, apparently caused by the R298A mutation, the C-terminus flips in the opposite direction towards the helical domain so that Val303 side chain, the last residue visible in the electron density, is as close as 4 Å to Ser1 side chain of the same protomer (Figs. 6f and S6). Although C-terminal residues do not make hydrogen bonds at the dimer interface, they interact through hydrophobic interactions with the other protomer in MPro^WT^. These interactions are lost in MPro^M^-GC373 due to the drastic conformational change of the C-terminus in the double mutant.

## Discussion

All precursor analogues used in this study autocatalyze the hydrolytic cleavage at the N-terminus (nsp4/nsp5 site) of MPro including the monomeric ^(−25)^MPro^1–199^ which lacks the entire helical domain (domain III). *E. coli* expression of constructs comprising MPro^WT^ fused to native cleavage sites at the N- and C-termini results in processed mature MPro^WT^ without observing any full-length or intermediate products, consistent with previous reports^9,11,28^. Similarly, full-length precursors are not observed upon expression of Precursor^R298A^ and ^(−102)^MPro^R298A^. In contrast, full-length precursors accumulate in *E. coli* upon expression of Precursor^E290A^, Precursor^M^, ^(−102)^MPro^E290A^, ^(-102)^MPro^M^, ^(−25)^MPro^M^, and ^(−25)^MPro^199^ that undergo time-dependent autoprocessing reaction at the N-terminus (Figs. 1, 2 and S2). We isolated the full-length precursors and the products of the autoprocessing reactions from *E. coli*. The results in Table 2 show the effect of mutations on the kinetic parameter k_cat_/K_m_, binding constants to the known covalent inhibitor GC373, and the dimer dissociation constants. Mutations E290A and R298A affect both k_cat_/K_m_ and K_dimer_ to a different extent. MPro^R298A^ exhibits a k_cat_/K_m_ of 0.31 μM^−1 ^min^−1^, i.e., about half that of MPro^WT^, and a K_dimer_ of 7.13 μM, i.e., about 5.5 times larger than that of MPro^WT^. E290A mutation has a much larger effect on k_cat_/K_m_ and K_dimer_ compared to the R298A mutation. The observed k_cat_/K_m_ for MPro^E290A^ is within the range of the double mutant MPro^M^. The observed K_dimer_ of 353 μM is about 19 times smaller than that of MPro^M^ and about 270 times larger than that of MPro^WT^ (Table 2). These results are consistent with the observed accumulation profile of the precursor in *E. coli* and highlight the role of dimer formation in the autoprocessing reaction. Precursor comprising MPro^R298A^ does not accumulate in *E. coli* and only the fully processed mature enzyme upon cleavages at both termini is observed. While the intrinsic catalytic activity (k_cat_/K_m_) of MPro^E290A^ is nearly identical to that of MPro^M^, the precursor comprising MPro^E290A^ undergoes stepwise autoprocessing at both termini at a much faster rate because of its smaller K_dimer_. In contrast, precursors comprising MPro^M^ and MPro^1–199^ accumulate and undergo autoprocessing in the monomeric form at a much slower rate in cells which allows their purification for in vitro reactions. It is intriguing, however, that the monomeric precursor comprising MPro^M^ with only the nsp5/nsp6 cleavage site, even in its dimer form, failed to promote cleavage at the C-terminus of MPro^M^ despite the accessibility for cleavage of this site by provided MPro^WT^.

Similarly, E290A and R298A mutations affect the binding of the transition state analog GC373 to varying extent. While the binding constant of GC373 to MPro^R298A^ is within the experimental error to that of MPro^WT^, MPro^E290A^ is 23 times larger relative to MPro^WT^ and comparable to that of MPro^M^. Furthermore, the mutant enzymes bind the aldehyde inhibitor GC373 with a binding constant inversely proportional to k_cat_/K_m_ which is consistent with previously reported studies [see Table 2 and ref. ^11,13^].

Room temperature crystal structure of Mpro^M^ shows that E290A and R298A mutations destabilize the dimer by excluding all direct hydrogen bonding between the protomers in the vicinity of residue 290 and retaining one hydrogen bond near residue 298. In the presence of the inhibitor, the dimer is stabilized through hydrophobic interactions, bridged by hydrogen bonding through water molecules and a H-bond between Gln299 of protomer A with Ser139 of protomer B. Destabilization of the dimer form is reflected in the catalytic activity, the K_dimer_, binding of the covalent inhibitor GC373 (Table 2) as well as the accumulation of the full-length precursor upon expression in *E. coli*. Both mature MPro mutants, MPro^E290A^ and MPro^R298A^, follow second-order kinetics in protein concentration for catalyzing the hydrolysis of the peptide substrate (Fig. 2c, d) indicating that the observed catalytic activity is that of a dimer, like that of MPro^WT^ and MPro^M^ and in contrast to that of the monomeric catalytic activity of MPro^1–199^ ^11^. Recently, we reported the room temperature X-ray structures of MPro^1–199^ and MPro^1–196^-GC373 complexes^11^. The structure of the monomeric MPro^1–199^ is nearly identical to that of the dimeric wild-type except for the oxyanion-loop being in an unwound conformation (inactive E-state), whereas MPro^1–196^-GC373 covalent complex displays a wound oxyanion loop (active E*-state) typical of the dimeric form of inhibitor-free mature MPro. The observed catalytic activities of MPro^M^ and MPro^1–199^ indicated that the E-E* equilibrium is dynamic in which the E state predominates in the monomer while the E* is the catalytically active species and favored in the dimer^11–13^.

Although we were unable to isolate Precursor^E290A^ in sufficient purity and quantity for in vitro analysis due to the relatively fast autoprocessing reaction, small-scale isolation of the full-length precursor and its products enabled verification by mass spectrometry. Precursor^E290A^ exhibits time-dependent autoprocessing *via* initial cleavage at the N-terminus to produce MPro^E290A-IP^ followed by the cleavage at the C-terminus to produce mature MPro^E290A^ (Fig. 1e-g). This order of cleavage is consistent with the observed cleavage order of a construct comprising 10 amino acid of the native flanking sequence at the N- and C-termini of MPro of SARS-CoV having an active site C145A mutation catalyzed intermolecularly by mature MPro^WT^ ^29^.

As indicated above, ^(−102)^MPro^M^ and ^(−25)^MPro^199^ undergo time-dependent autoprocessing at the N-terminus to produce MPro^M^ and MPro^199^ in vitro (Fig. 3). The results in Table 1 show that the percent conversion of the precursor to products at a given time is independent of the initial protein concentration indicating that the reactions follow first-order kinetics, i.e., unimolecular. The percent conversion of the two precursors at a given time are similar, if not identical. Thus, the cleavage at the N-terminus of MPro occurs *via* an intramolecular mechanism from a monomer in these two model precursors. Importantly, the addition of GC373 to the precursor analogue, ^(−25)^MPro^M^, increases the rate of autoprocessing and the increase in rate of autoprocessing is dependent on the GC373 concentration. This attribute is similar to that reported for the activation of MPro^M^ by GC373 and can be explained by a similar mechanism^13^. In that mechanism, MPro^M^ is predominantly in the monomer form with an oxyanion loop in the E conformation. Upon binding GC373, MPro^M^ forms a dimer concomitant with the reorganization of the oxyanion loop to the active E* conformation. In the presence of the equimolar amount of protein and GC373, three major protein species are present in equilibrium: monomer, dimer with one of the active sites occupied by GC373, and a dimer with both active sites occupied by GC373. The increase in catalytic activity is attributed to the presence of the population of a dimer with one active site occupied by GC373, leaving the other active site with an oxyanion loop in the active E* conformation for catalytic function. Thus, the increase in the reaction rate of ^(−25)^MPro^M^ autoprocessing in the presence GC373 must be due to the formation of a dimer. Accordingly, the autoprocessing of the model precursor is initiated by N-terminal intramolecular cleavage from a monomer or a dimer. The enhanced dimer stability in the context of a wild-type precursor, relative to MPro^M^, suggests that the initial cleavage at the N-terminus most likely occurs from the dimer because it is more reactive than the monomer. This conclusion is in accordance with the precursor accumulation profile in *E.coli* described above.

N-terminal cleavage of ^(−102)^MPro^M^ to produce MPro^M^ is accompanied by an increase in catalytic activity as well as the binding affinity to GC373 increasing by about 3-fold (Table 2, Fig. S4c). As previously reported, the observed catalytic activity of MPro^M^ is that of a dimer indicating that N-terminal cleavage will favor dimer formation. The involvement of the N-finger in dimer formation has been examined in detail by structural and mutational studies. Deletion and mutational studies of the N-finger residues 1–9 show drastic effect on dimer formation and catalytic activity, relative to MPro^WT^, consistent with the critical interfaces involving the N-finger with domains II and III^8,11^. In recent studies, the effect of flanking nsp4 residues, contrary to deleting the N-terminal residues of MPro, on K_dimer_ was also examined^12^. The active site H41A mutation to restrict N-terminal autoprocessing enabled the analyses of such precursor mimetics. Even the addition of six residues of the native nsp4 sequence to mature MPro^H41A^ (^(−6)^MPro^H41A^) increases the K_dimer_ by >85-fold. Structural studies revealed asymmetry, semi-open conformation and disorder of the terminal residues of the dimeric ^(−6)^MPro^H41A^ ^12^. Thus, the product of the autoprocessing reaction to liberate the free N-terminus of MPro is critical for dimer formation.

The product MPro^M-IP^ resulting from the autoprocessing of Precursor^M^ containing the nsp5/nsp6 cleavage site at the C-terminus of MPro^M^ exhibits catalytic activity, which is that of a monomer and is activated and inhibited by GC373 like that of mature MPro^M^. Activation by GC373 is concomitant with dimer formation. However, it does not autocatalyze the nsp5/nsp6 junction indicating that the monomer is incapable of this cleavage either in the absence, or presence of GC373 in its dimer form. But treatment of the monomeric MPro^M-IP^ with MPro^WT^ produces MPro^M^ indicative of the site being accessible for cleavage in its monomeric form. Failure of MPro^M-IP^ to process its own C-terminus could be due to its inability to form a dimer. The requirement of a dimer for C-terminal cleavage is consistent with the observations in crystal structures showing the C-terminal residues of one dimer of MPro^C145A^ bound to the active site of a second dimer^30–32^. It is unclear as to why MPro^M-IP^ fails to undergo C-terminal cleavage in the presence of equimolar amount of GC373 when both monomer and the enzymatically active dimer coexist. This may point to the dynamics of this system which is incapable of forming a productive reaction complex. It is worth noting, however, that lack of C-terminal autoprocessing is limited only to MPro^M-IP^ having both mutations E290A and R298A, whereas MPro^E290A-IP^ exhibits C-terminal cleavage likely due to its ability to form a significant population of dimer even in the absence of inhibitor GC373.

**Fig. 7:**
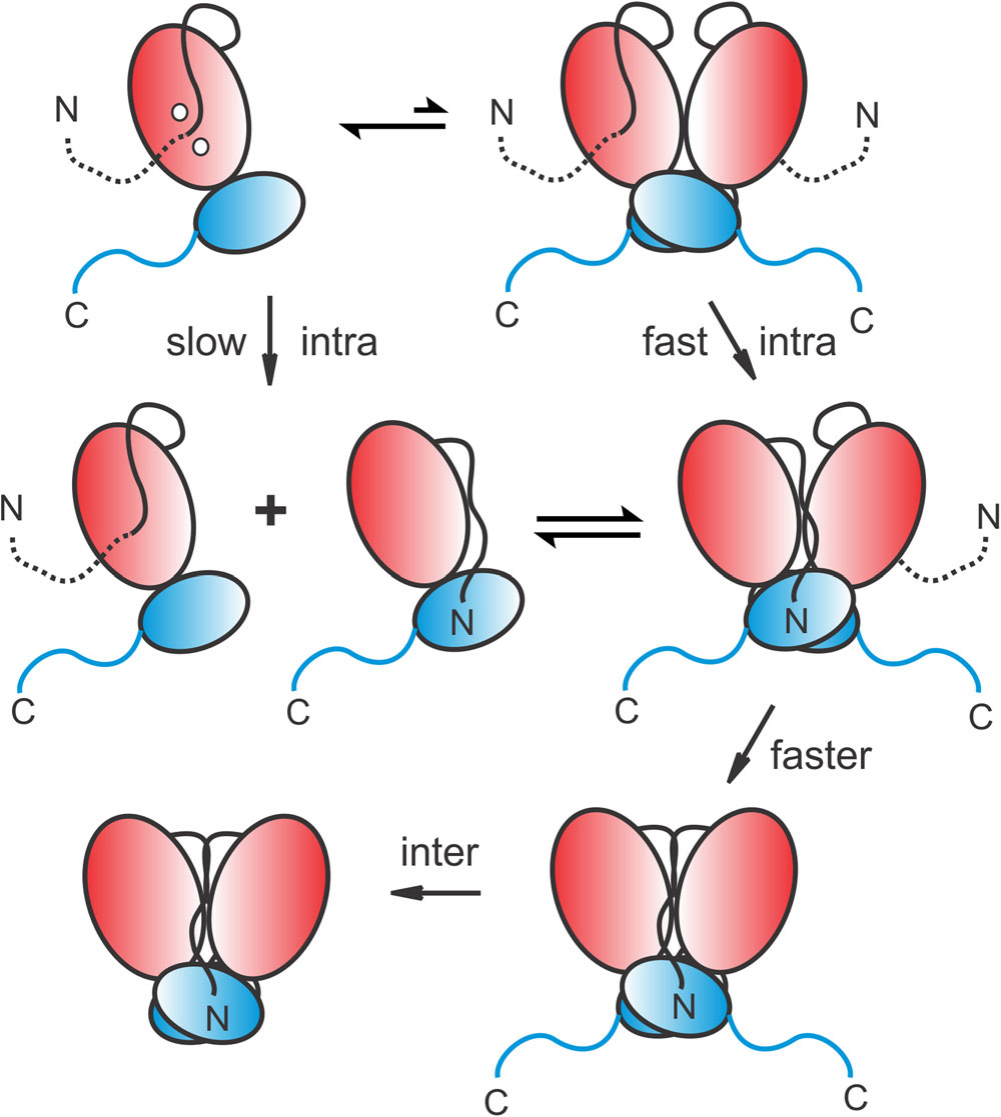
Proposed mechanism of MPro autoprocessing. Catalytic (domains I and II) and helical (domain III) regions are shown as red and blue ovals, respectively. Solid black lines denote the N-terminal residues (N-finger) of MPro. Dashed black and solid blue lines represent the truncated nsp4 and nsp6 regions flanking the N- and C-termini of MPro. Catalytic dyad H41 and C145 residues are indicated as white circles in the top left monomer cartoon.

Transient accumulation of Precursor^E290A^ and its conversion to MPro^E290A-IP^ and mature MPro^E290A^ indicates that cleavage at the N-terminus precedes the C-terminal cleavage. A proposed mechanism for MPro autoprocessing from a model precursor is shown in Fig. 7. In this mechanism, the MPro region of the monomeric precursor exhibits a tertiary fold like that of the mature subunit with an oxyanion loop predominantly in the E-state. This inactive state is in equilibrium with a minor population having the active E* conformation, which undergoes slow unimolecular N-terminal autoprocessing. The monomeric precursor is also in equilibrium with its homodimer. This is supported by the observation that the presence of N- and C-terminal flanking sequences increase the K_dimer_ significantly but do not exclude the precursor from becoming a dimer. In the transient precursor dimer, oxyanion loop dynamics favor an E* conformation leading to a faster rate of cleavage of the N-terminal site than that of the monomeric precursor. Results presented here show that the monomer and dimer precursor forms can autocatalyze N-terminal cleavage and that the dimer undergoes significantly faster reaction, compared to the monomer. The observed first-order kinetics of the monomeric model precursors indicate that the cleavage at the N-terminus proceeds *via* an intramolecular mechanism. N-terminal cleavage of a monomer or a dimer would lead to an increase in the dimer population as the newly formed free N-finger adopts a native-like interface with the second protomer. A heterodimer comprising a free N-terminus is likely to be more stable than the precursor homodimer and hence, being more reactive leads to an even faster rate of cleavage of the second N-terminus resulting in further stabilization of the dimer interface and decrease in K_dimer_. It is likely that the precursor protomer containing the N-terminal flanking sequence is responsible for the cleavage of its own N-terminus because it conforms with the correct orientation for substrate binding visualized in structures^33,34^. Since the N-terminal flanking sequence has a larger effect on the K_dimer_ as well as the GC373 binding constant (Table 2 and Fig. S4c) than the C-terminal flanking sequence^29^, upon N-terminal cleavage, a more stable population of homo- and heterodimers favoring the E* conformation of the oxyanion loop leads to the processing of the C-terminal nsp5/nsp6 site *via* an intermolecular mechanism to release the mature enzyme.

The above mechanism of activation is consistent with reports on the self-cleavage of picornaviral replicase precursors^35,36^ and early cleavages of 3CL protease of coronavirus MHV-A59^37^. Both nsp4 and nsp6 are shown to have multiple transmembrane domains and thus, MPro is suggested to be anchored on both sides to membranes. The resulting major products nsp5-nsp10 and nsp5-nsp16 generated from pp1a and pp1ab, respectively, by cleavage at the N-terminus may be transported through membrane association^17^ for processing of the nsp5/nsp6 site through a concentration-dependent intermolecular mechanism at a later stage of viral polyprotein maturation.

Also, the proposed mechanism of MPro autoprocessing is strikingly similar to that of the dimeric aspartic acid protease of HIV-1 from its precursor^1,3,38^. The main difference lies in the cleavage at the N-terminus of HIV protease occurring from the dimer form because each of the monomer possesses only half of the catalytic residues of the active site. Thus, it appears that intramolecular cleavage at the N-terminus followed by intermolecular cleavage at the C-terminus is a general mechanism for autoprocessing of proteases of RNA viruses from their polyprotein precursor.

## Methods

### Construction and designation of MPro constructs

Expression and purification of MPro^WT^ (GenBank ID: MN908947.3), MPro^M^ and MPro^1–199^ were described before^11,13,39^. New constructs prepared for this work are: Precursor^WT^, Precursor^M^ and its single mutants, precursor ^(−102)^MPro^WT^, precursor ^(−102)^MPro^M^ and its single mutants, precursor ^(−25)^MPro^1–199^, mature MPro^R298A^ and mature MPro^E290A^. All constructs were synthesized and cloned into pJ414 vector (ATUM, Newark, CA). Designations and amino acid sequences of the constructs are listed in Figure S1.

### Expression and purification

Plasmids were transformed into BL21-DE3 cells (Agilent) and induced for expression at 0.7–0.8 optical density with 1 mM isopropyl β-d-1-thiogalactopyranoside, typically for 3 h at 37 °C. Proteins were purified from the cell lysate by nickel-affinity chromatography (NAC, step 1). The bound fraction was subjected to isocratic fractionation on Superose-12 column (step 2, Cytiva Life Sciences) in a final buffer of 25 mM Tris-HCl, pH 7 or 7.2, 150 mM NaCl and 1 mM TCEP (buffer A). Peak fractions were pooled and concentrated to the desired concentration using Amicon Ultra – 15 or 0.5 ml centrifugal filters (Merck Millipore Ltd.) and stored in aliquots at −30 °C and for long-term storage at −80 °C. Purity was verified both by SDS-PAGE on 4–20% gradient mini-protean TGX precast gel (Bio-Rad) and reverse-phase liquid chromatography with in-line electrospray ionization mass spectrometry^11^. Protein concentrations were measured before storage and prior to the experiment at least in duplicate based on the extinction coefficient (Fig. S1) at 280 nm.

Purifications of mature MPro^R298A^ and MPro^E290A^ were carried out with an additional HRV-3C protease cleavage step to remove the C-terminal 6His-tag as described previously for MPro^WT^ and MPro^M^ ^11^. To isolate precursors ^(−102)^MPro^M^ and ^(−25)^MPro^1–199^, cell cultures (<100 ml) were induced for <30 min and chilled immediately prior to harvesting. Cell pellets were subjected to the same purification scheme as above not exceeding a total time of 4 h at 4 °C due to autoprocessing at native conditions. The final column step on Superose-12 was carried out in buffer B (25 mM Tris-HCl, pH 7, 50 mM NaCl and 1 mM TCEP). The full-length protein, representing only a small fraction of the total load on the column, with minimal contamination of the products were pooled and stored in aliquots at −70 °C.

### Autoprocessing

Time course of the autoprocessing reaction was carried out as follows. Cells (12 ml) were harvested at the indicated time points, chilled on ice, and subjected to NAC on spin columns. Equal volumes of the bound fraction were analyzed by SDS-PAGE. But for side-by-side comparison of the flow through (FT) and bound (B) fractions and monitoring the autoprocessing reactions in vitro (Tables S1, S2), equal amounts of proteins^11^ were used for gel fractionation. Molecular weight markers (in kDa) are shown for all gel panels except in few panels when a protein of similar mass (Figs. 2 and S1) or protein from the same stock solution are analyzed (Fig. 4). Uncropped gel images of Figs. 1–4 and S2 are shown in Fig. S7.

### Enzyme kinetics

Activity assays using the FRET substrate Dabcyl-KTSAVLQ/SGFRKM-E(Edans)-NH_2_, where (/) denotes the scissile peptide bond, were performed in a total volume of 100 µl in buffer B (25 mM Tris-HCl, pH 7, 50 mM NaCl and 1 mM TCEP) at 28 °C as previously described^9,13^. Assays were carried out with equimolar amount of GC373 and enzyme and varying the substrate concentration from 5–200 µM. k_cat_/K_m_ was calculated from plots of 1/v *vs* 1/S and the GC373 binding constant was determined by ITC. Plots of rate *vs* the square of the protein concentration at 200 μM were linear, and the slope of the line is 2 _kcat_[S]/K_dimer_(K_m_ + [S]). Since the K_m_ value is about the same as the substrate concentration used, the slope of the line is simplified to k_cat_/K_m_K_dimer_ that allowed the calculation of K_dimer_. For details, see reference ^13^. The substrate was custom synthesized (Biomatik, Ontario, Canada), and GC376 was purchased from Selleckchem, Houston, TX.

### Sedimentation velocity analytical ultracentrifugation (SV-AUC) and Isothermal titration calorimetry (ITC)

SV-AUC (in buffer B at 25 °C) and ITC in buffer C (25 mM Tris-HCl, pH 7.2, 20 mM NaCl and 1 mM TCEP) at 28 °C were carried out as described in references ^11–13^.

### Protein crystallization and room-temperature X-ray crystallography

MPro^M^ protein sample was concentrated to 7 mg/ml. GC376 stock was prepared at 10 mM GC376 in 25 mM Tris-HCl, pH 7.2, 20 mM NaCl and 1 mM TCEP for crystallization purposes and stored at −30 °C. GC376 is converted to the reactive aldehyde GC373 when mixed with an aqueous solution. For co-crystallization, MPro^M^ was mixed with GC376 at 1:5 molar ratio and allowed to incubate at room temperature for at least 30 min before setting up crystal trays. Crystals of GC373-bound MPro^M^ were grown at 14 °C by sitting drop vapor diffusion methodology with 18–21% PEG3350, 0.1 M Bis-Tris pH 6.5, or pH 7.0 (1 mL) as the precipitant solution. Crystallization drops of 20 µL at 1:1 ratio were seed struck using the crystals of the native MPro in complex with a covalent ligand NBH2 as described in references ^26,39^. The crystals suitable for X-ray diffraction measurements were mounted in MiTeGen (Ithaca, NY) room-temperature capillary setups for data collection.

Room temperature X-ray crystallographic data were collected on a Rigaku HighFlux HomeLab instrument equipped with a MicroMax-007 HF X-ray generator, Osmic VariMax optics, and a DECTRIS Eiger R 4 M hybrid photon counting detector. X-ray diffraction data were integrated using the CrysAlis Pro software suite (Rigaku Inc., The Woodlands, TX), then reduced and scaled using Aimless^40^ from the CCP4 suite^41^. Structures were solved by molecular replacement using Phaser^42^. MPro^WT^-GC373 complex structure (PDB code 7UKK)^11^, was used as a search model to solve the structure of MPro^M^-GC373 complex. The model was iteratively refined with *phenix.refine* from the PHENIX suite^43^ and COOT^44^. Geometry validation was aided by Molprobity^45^. GC373 restraints were generated with eLBOW^46^ using geometry optimized by quantum chemical calculations in Gaussian16 at B3LYP/6-31 g(d,p) level of theory^47^. Final data collection and refinement statistics can be found in Table 3.

### Statistics and reproducibility

Expressed proteins were verified both by DNA sequencing and mass spectrometry. The reproducibility of enzyme kinetics was tested at least 2–3 times with freshly prepared enzyme and stock solutions of the substrate and inhibitor. Once this was determined to provide consistent reaction rates within an error limit of 5%, the final experiment for the data displayed in the manuscript was carried out in duplicate and 4 reads per well for each time point. The mean of the data points was used for fitting. The same stock solutions of enzyme and inhibitor were used for SV-AUC and ITC analyses to determine the dimer dissociation constant (K_dimer_) and the binding constant of the inhibitor to the enzyme (K_d_), respectively. K_dimer_, and K_d_ were determined with multiple protein constructs (Fig. S1) and concentrations. Each ITC experiment was carried out with a minimum of 20 injections. The apparent dimer dissociation constants were determined by Lamm equation modeling of the absorbance and interference data. X-ray diffraction data and refinement statistics are shown. Gel images are best representative for each of the construct analyzed.

### Reporting summary

Further information on research design is available in the Nature Portfolio Reporting Summary linked to this article.

### Supplementary information

Supplementary Information

Description of Additional Supplementary Files

Supplementary Data 1

Supplementary Data 2

Reporting Summary

## Data Availability

The structure and corresponding structure factors have been deposited into the protein data bank with the PDB accession code 8FI9 for MPro^M^/GC373 complex. Source data files are provided in Supplementary Data 1, 2, and Supplementary information. All other data that support this study are available from the corresponding authors upon reasonable request.
