# Supplementary material for: Insights into the mechanism of SARS-CoV-2 main protease autocatalytic maturation from model precursors
Source: Commun Biol. 2023 Nov 13;6:1159. doi: 10.1038/s42003-023-05469-8 (PMC10643566; doi:10.1038/s42003-023-05469-8)
Supplement: Supplementary file 1 — Supplementary Information [file 42003_2023_5469_MOESM1_ESM.pdf]

**Supplementary Information**

# Insights into the mechanism of SARS-CoV-2 main protease autocatalytic maturation from model precursors

Annie Aniana,[1] Nashaat T. Nashed,[1] Rodolfo Ghirlando,[2] Leighton Coates,[3] Daniel W. Kneller,[4,5] Andrey Kovalevsky,[4*] John M. Louis[1*]

[1]*Laboratory of Chemical Physics, National Institute of Diabetes and Digestive and Kidney Diseases, National Institutes of Health, DHHS, Bethesda, MD 20892-0520, USA*

[2]*Laboratory of Molecular Biology, National Institute of Diabetes and Digestive and Kidney Diseases, National Institutes of Health, DHHS, Bethesda, MD 20892-0520, USA*

[3]*Second Target Station, Oak Ridge National Laboratory, 1 Bethel Valley Road, Oak Ridge, TN, 37831, USA*

[4]*Neutron Scattering Division, Oak Ridge National Laboratory, 1 Bethel Valley Road, Oak Ridge, TN, 37831, USA*

[5]*Present address: New England Biolabs, 240 County Road, Ipswich, MA 01938-2723, USA*

* Corresponding authors:
Andrey Kovalevsky: kovalevskyay@ornl.gov, John M. Louis: johnl@niddk.nih.gov

**Contents**

Table S1 and S2……………………………………………………………………………......3
Figure S1…………………………………………………………………………………………4
Figure S2…………………………………………………………………………………………7
Figure S3…………………………………………………………………………………………8
Figure S4…………………………………………………………………………………………9
Figure S5………………………………………………………………………………………..10
Figure S6………………………………………………………………………………………..11
Figure S7………………………………………………………………………………………..12
References………………………………………………………………………………………17

Table S1. **Reaction set-up for monitoring the time course of the autoprocessing reaction as a function of decreasing precursor $^{(-102)}$MPro$^{M}$ concentration in buffer B at pH 7 and 28 °C.**

| $^{(-102)}$MPro$^{M}$ reaction concentration (µM) | Reaction volume (µL) | Volume (µl) drawn/time point | Buffer B (µl) | Gel sample buffer (µl) | Volume (µl) loaded / lane | Amount (µg) loaded / lane |
|---|---|---|---|---|---|---|
| 96 (4.46 mg/ml) | 10 | 1.5 | 27 | 12.9 | 16 | 2.6 |
| 4.8 | 162.5 | 25 | 0 | 7 | 16 | 2.8 |

Table S2. **Reaction set-up for monitoring the time course of the autoprocessing reaction as a function of decreasing precursor $^{(-25)}$MPro$^{1-199}$ concentration in buffer B at pH 7 and 28 °C.**

| $^{(-25)}$MPro$^{1-199}$ reaction concentration (µM) | Reaction volume (µL) | Volume (µl) drawn/time point | Buffer B (µl) | Gel sample buffer (µl) | Volume (µl) loaded / lane | Amount (µg) loaded / lane |
|---|---|---|---|---|---|---|
| 96 (2.57 mg/ml) | 30 | 2.8 | 17.2 | 10 | 15 | 3.6 |
| 48 | 36.4 | 5.6 | 14.4 | 10 | 14 | 3.4 |
| 9.6 | 182 | 28 | 0 | 12 | 18.7 | 3.4 |

Figure S1.

**Amino acid sequence of MPro constructs.**

---

## Precursor$^{WT}$, Precursor$^{M}$ and its single mutants

```
           -25                  -7        1             14          24
GSSHHHHHHS GNDFSNSGSD VLYQPPQTSI TSAVLQSGFR KMAFPSGKVE GCMVQVTCGT
         34         44         54         64         74         84
TTLNGLWLDD VVYCPRHVIC TSEDMLNPNY EDLLIRKSNH NFLVQAGNVQ LRVIGHSMQN
         94        104        114        124        134        144
CVLKLKVDTA NPKTPKYKFV RIQPGQTFSV LACYNGSPSG VYQCAMRPNF TIKGSFLNGS
        154        164        174        184        194        204
CGSVGFNIDY DCVSFCYMHH MELPTGVHAG TDLEGNFYGP FVDRQTAQAA GTDTTITVNV
        214        224        234        244        254        264
LAWLYAAVIN GDRWFLNRFT TTLNDFNLVA MKYNYEPLTQ DHVDILGPLS AQTGIAVLDM
        274        284      A   294    A     304 +1   GB1
CASLKELLQN GMNGRTILGS ALLEDEFTPF DVVRQCSGVT FQSAVMQYKL ILNGKTLKGE

TTTEAVDAAT AEKVFKQYAN DNGVDGEWTY DDATKTFTVT ELEHHHHHH
```

**Precursor$^{WT}$**
**Calculated molecular weight:** 45173.1
Ext. coefficient: 41960 (280 nm)

**Precursor$^{M}$**
**Calculated molecular weight:** 45029.9
Ext. coefficient: 41960 (280 nm)

**MPro$^{M\text{-}IP}$**
**Calculated molecular weight:** 41180.9
Ext. coefficient: 40680 (280 nm)

**Precursor$^{R298A}$**
**Calculated molecular weight:** 45087.9
Ext. coefficient: 41960 (280 nm)

**Precursor$^{E290A}$**
**Calculated molecular weight:** 45115
Ext. coefficient: 41960 (280 nm)

**MPro$^{E290A\text{-}IP}$**
**Calculated molecular weight:** 41266
Ext. coefficient: 40680 (280 nm)

---

## Precursor $^{(-102)}$MPro$^{WT}$, $^{(-102)}$MPro$^{M}$ and its single mutants

```
-102            -85        -75        -65        -55        -45
SGKRRVVFNG VSFSTFEEAA LCTFLLNKEM YLKLRSDVLL PLTQYNRYLA LYNKYKYFSG
        -35        -25        -15         -5    1              16
AMDTTSYREA ACCHLAKALN DFSNSGSDVL YQPPQTSITS AVLQSGFRKM AFPSGKVEGC
         26         36         46         56         66         76
MVQVTCGTTT LNGLWLDDVV YCPRHVICTS EDMLNPNYED LLIRKSNHNF LVQAGNVQLR
```

```
         86        96        106       116       126       136
VIGHSMQNCV LKLKVDTANP KTPKYKFVRI QPGQTFSVLA CYNGSPSGVY QCAMRPNFTI
        146       156       166       176       186       196
KGSFLNGSCG SVGFNIDYDC VSFCYMHHME LPTGVHAGTD LEGNFYGPFV DRQTAQAAGT
        206       216       226       236       246       256
DTTITVNVLA WLYAAVINGD RWFLNRFTTT LNDFNLVAMK YNYEPLTQDH VDILGPLSAQ
        266       276       286   A     296 A       306
TGIAVLDMCA SLKELLQNGM NGRTILGSAL LEDEFTPFDV VRQCSGVTFQ GPHHHHHH
```

**$^{(-102)}$MPro$^{WT}$**
**Calculated molecular weight:** 46512.2
Ext. coefficient: 41390 (280 nm)

**$^{(-102)}$MPro$^{M}$**
**Calculated molecular weight:** 46369.1
Ext. coefficient: same as above

**$^{(-102)}$MPro$^{R298A}$**
**Calculated molecular weight:** 46427.1
Ext. coefficient: same as above

**$^{(-102)}$MPro$^{E290A}$**
**Calculated molecular weight:** 46454.2
Ext. coefficient: same as above

**-------------------------------------------------------------------------------------------------------------------**

**$^{(-25)}$MPro$^{1\text{-}199}$**

```
                     -25                   -6      1             15
GSSHHHHHHS SGENLYFQGS NDFSNSGSDV LYQPPQTSIT SAVLQSGFRK MAFPSGKVEG
         25        35        45        55        65        75
CMVQVTCGTT TLNGLWLDDV VYCPRHVICT SEDMLNPNYE DLLIRKSNHN FLVQAGNVQL
         85        95        105       115       125       135
RVIGHSMQNC VLKLKVDTAN PKTPKYKFVR IQPGQTFSVL ACYNGSPSGV YQCAMRPNFT
        145       155       165       175       185       195
IKGSFLNGSC GSVGFNIDYD CVSFCYMHHM ELPTGVHAGT DLEGNFYGPF VDRQTAQAAG
  199
TDTT
```

**Calculated molecular weight:** 26760.2
Ext. coefficient: 18490 (280 nm)

Figure S1. **Amino acid sequence of recombinant MPro constructs used in this study and their designations.** Non-native residues flanking the sequences are underlined. Mutation sites are shown in red. Theoretical mass of the purified protein is indicated below the sequence of the corresponding construct. $^{(-25)}$ and $^{(-102)}$ denote 25 and 102 amino acids of the C-terminal residues of nsp4, flanking the N-terminus of nsp5 (MPro), appended to MPro. $^{(+3)}$-GB1 denotes 3 N-terminal residues of the nsp6 sequence (SAV) followed by 56 residues of GB1 and 6His-tag. The 6H-tag at the C-terminus of Precursor$^{WT}$ and its analogues enables isolation by Nickel-Affinity Chromatography (NAC) and characterization of the intermediate precursor (MPro$^{M\text{-}IP}$) containing the C-terminal flanking sequence, which results from N-terminal cleavage at the nsp4/nsp5 site. As the size difference between the two is small (4.9 kDa), excluding the 6H-tag in construct $^{(-25)}$MPro$^{1\text{-}199}$ at its C-terminus enables purification of the full-length precursor exclusively without

the processed product MPro$^{1-199}$ by NAC for studies to determine the kinetic order of the reaction as described in Figure 3. Construct $^{(-25)}$MPro$^{1-199}$ contains a 6H-tag and a TEV protease cleavage site preceding 25 amino acids (-25) of the nsp4 sequence to enable purification of the precursor without the processed MPro$^{1-199}$. MPro$^{1-199}$ does not cleave the TEV site, evident from the isolation of full length $^{(-25)}$MPro$^{1-199}$ by NAC (Fig. 3) and mass spectrometry. Based on the simple rule that when the penultimate residue is Gly or Ser, in all our constructs, the N-terminal methionine is fully excised by methionyl-aminopeptidase as verified by mass spectrometry [1, 2].

Figure S2.

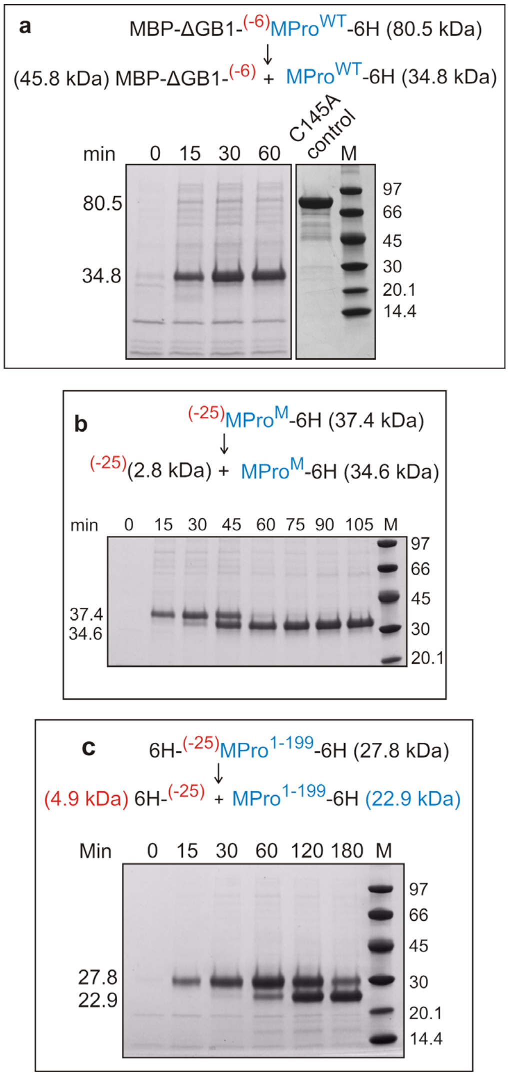


Figure S2. **N-terminal autoprocessing of MPro precursor mimetics in *E. coli*.** These panels are reproduced from our previous publication [3] solely for ease of comparison with the results presented in this work. The N-terminal cleavage site is indicated with a downward black arrow. The gels show the time course of the autoprocessing reaction of $MPro^{WT}$ **(a)**, $MPro^{M}$ **(b)** and $MPro^{1\text{-}199}$ **(c)** precursor constructs with flanking nsp4 sequences as indicated. Cells (12 ml) were harvested at the indicated time points, and equal volumes of the bound fractions following NAC were analyzed by SDS-PAGE. The precursor, products released upon cleavage at the N-terminus of MPro and molecular weight standards (M) are indicated in kDa.

Figure S3.

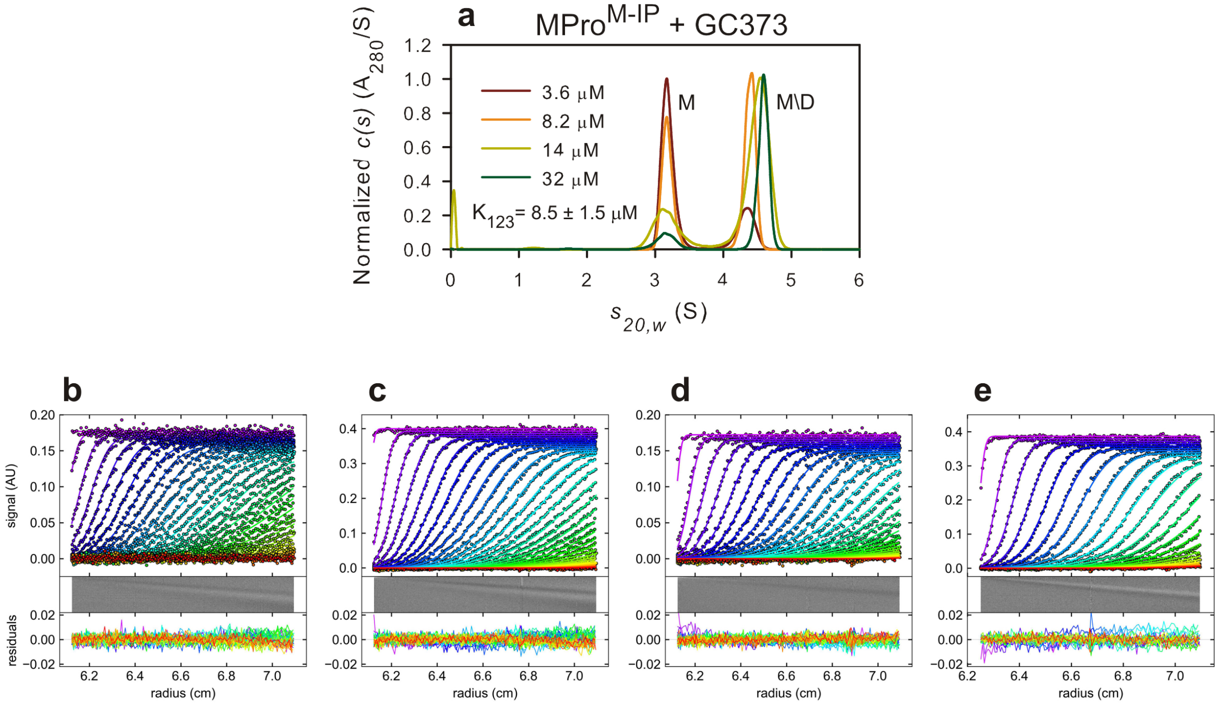


Figure S3. **Estimation of MPro$^{M\text{-}IP}$ dimer dissociation constant by SV-AUC. (a)** Normalized sedimentation velocity absorbance c(s) distributions for various concentrations of MPro$^{M\text{-}IP}$ in the presence of 2-fold molar excess GC373 support an inhibitor induced monomer-dimer self-association. M and M/D denote monomer and monomer/dimer equilibrium boundary, respectively. **(b-e)** Sedimentation data collected at 50,000 rpm and 25 °C over 7 hours at 3.6 to 32 µM MPro$^{M\text{-}IP}$ were analyzed globally in terms of a reversible monomer-dimer self-association using Lamm equation modeling (see legend to Fig. S5). The analysis returns a $K_{dimer}$ of 8.5 ± 1.5 µM.

Figure S4.

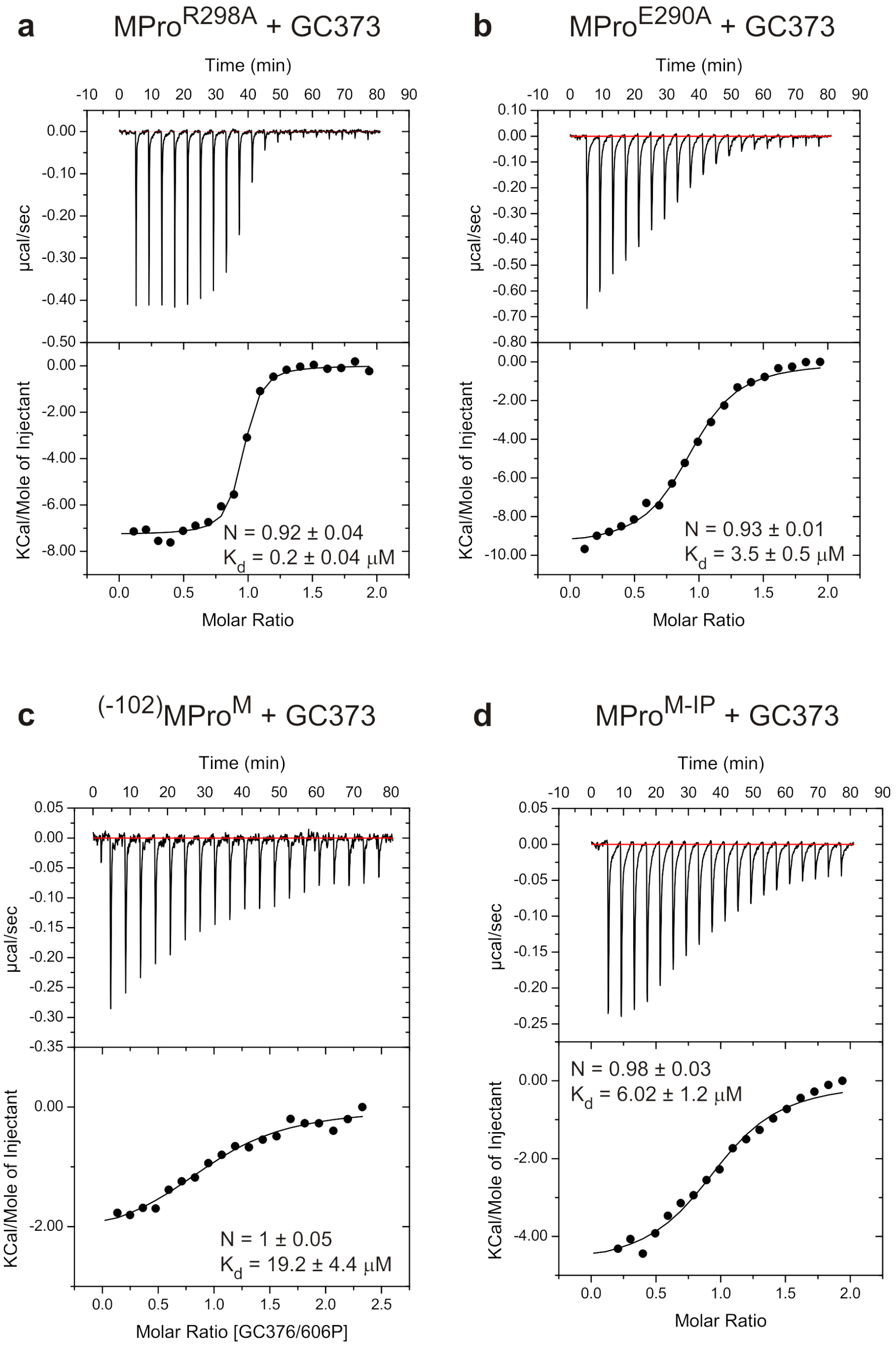


Figure S4. **Binding isotherms of inhibitor GC373 to mature MPro and its precursor mimetics.** Titrations were carried out in buffer C at 28 °C with **(a)** 48 µM MPro$^{R298A}$, **(b)** 90 µM MPro$^{E290A}$ **(c)** 100 µM $^{(-102)}$MPro$^{M}$ and **(d)** 80 µM MPro$^{M-IP}$ (in the cell) and GC373 (in the syringe) at 10 times the concentration of proein. Inhibitor dissociation constants are listed in Table 1.

Figure S5.

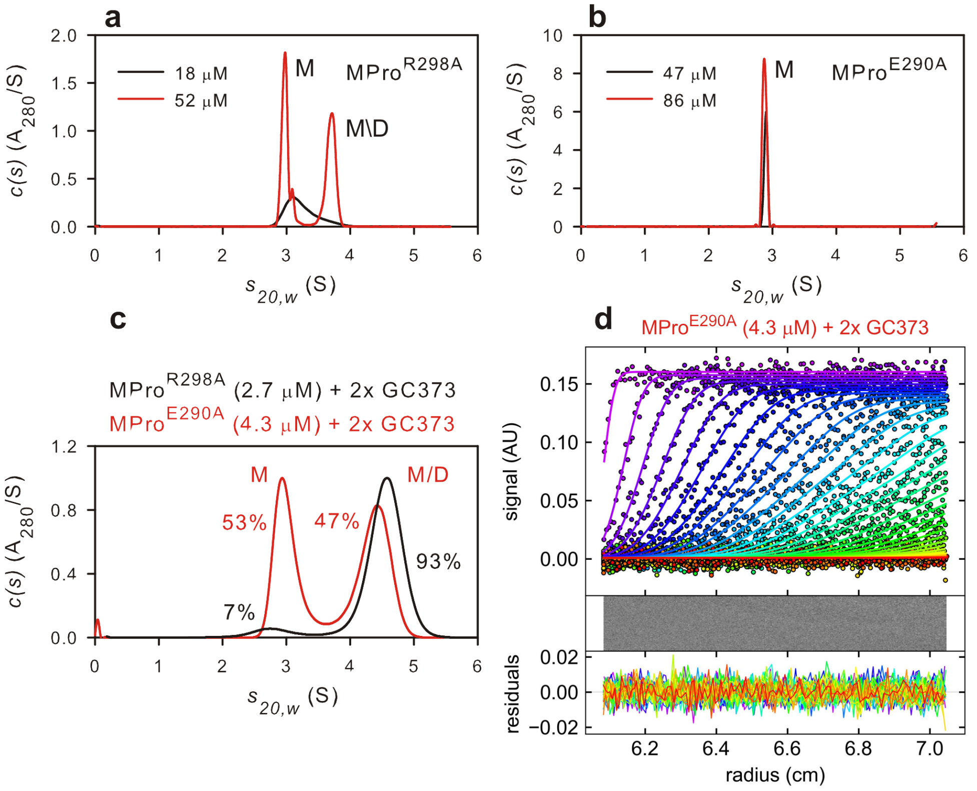


Figure S5. **Estimation of the dimer dissociation constant of MPro$^{R298A}$ and MPro$^{E290A}$ by SV-AUC.** Sedimentation velocity absorbance c(s) distributions at the indicated loading concentrations for **(a)** MPro$^{R298A}$, **(b)** MPro$^{E290A}$ and **(c)** MPro$^{E290A}$ in the presence of GC373. **(d)** Sedimentation velocity data collected at 50,000 rpm and 25°C in 3 mm pathlength cells with scans collected over 6 hours. The data were analyzed globally in terms of a reversible monomer-dimer self-association model using Lamm equation modeling. For clarity only every sixth scan and every third experimental data point are shown. Best-fits are represented by a solid line through the experimental points. A bitmap representation of the residuals to the best-fit, together with the combined residuals, are shown below each plot. The analysis returns a monomer-dimer dissociation constant ($K_d$) of 4.8 ± 0.6 μM for MPro$^{E290A}$ with GC373. Based on the loading concentration and dissociation constant, the dimer contribution (in monomer units) was determined to be 48% at 4.3 μM MPro$^{E290A}$ in the presence of 2x GC373. M and M/D denote monomer and monomer/dimer equilibrium boundary, respectively.

Figure S6.

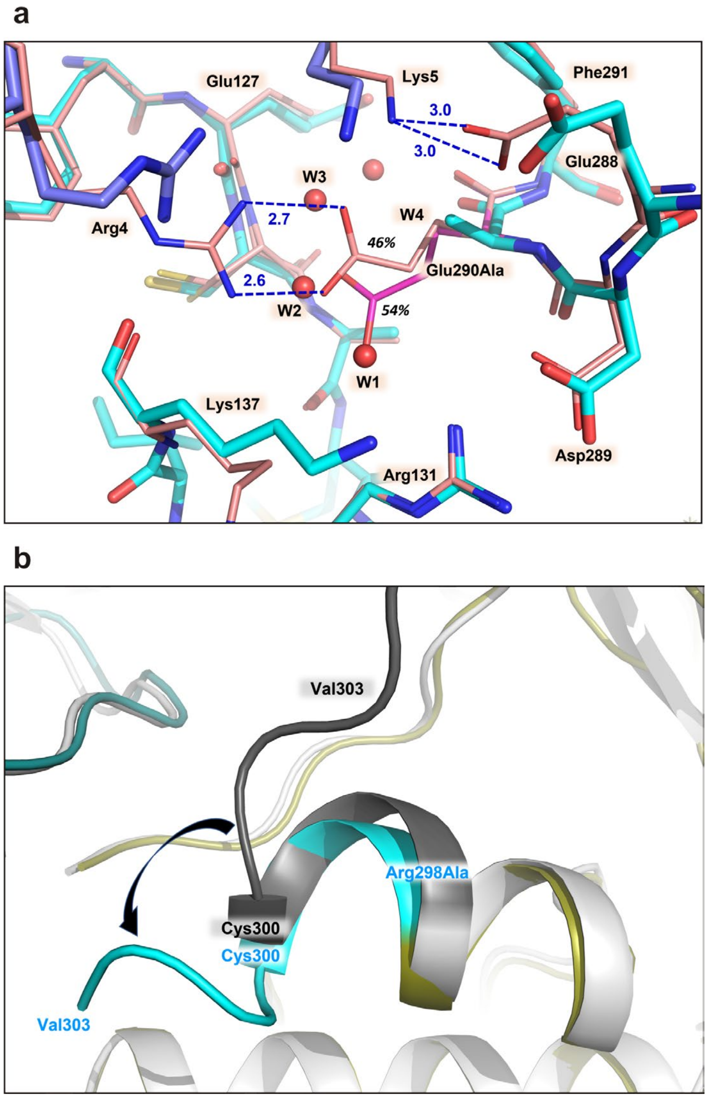


Figure S6. **Comparison of MPro$^{WT}$ and MPro$^{M}$ structures.** **(a)** Superposition of MPro$^{M}$ and MPro$^{WT}$ complexes with GC373 near E290A mutation site demonstrating the loss of the E290…R4' salt bridge. In MPro$^{WT}$-GC373 Glu290 has two alternate conformations with occupancies 46 and 54 %. **(b)** Superposition of MPro$^{M}$-GC373 (colored deep olive, this work, PDB ID 8FIG), MPro$^{WT}$-GC373 (colored salmon, PDB ID 7UKK [3]) and inhibitor-free MPro$^{WT}$ (colored gray, PDB ID 7JUN [4]) cartoon representations near the R298A mutation site shows a conformational reorientation of the C-terminal residues (black curved arrow). Residues beyond the site of mutation at position 298 are colored cyan for MPro$^{M}$ and dark gray for MPro$^{WT}$.

Figure S7. **Uncropped gel images of Fig. 1, 2, 3, 4 and S2.**

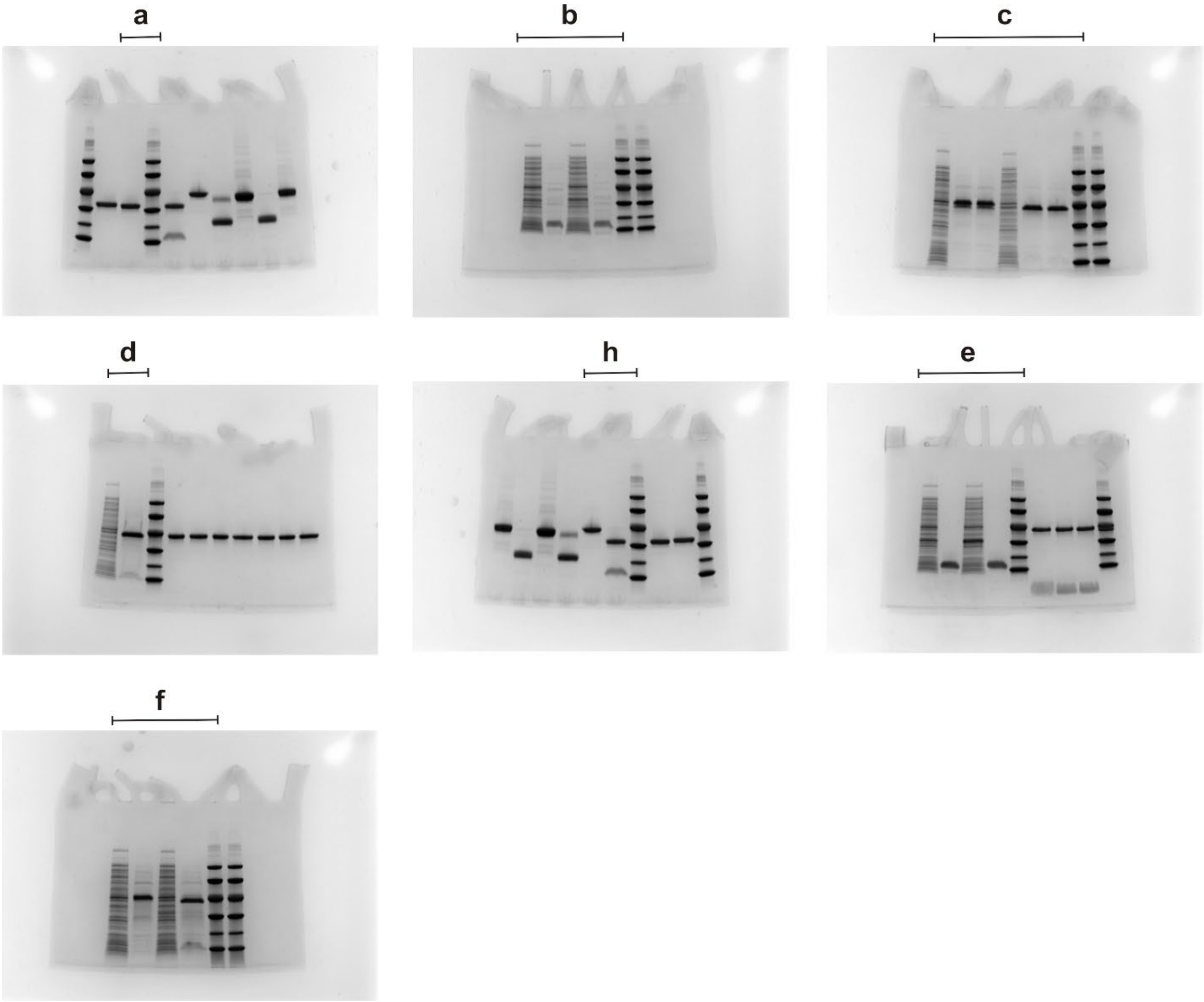


Figure S7. Uncropped gel images of Fig. 1.

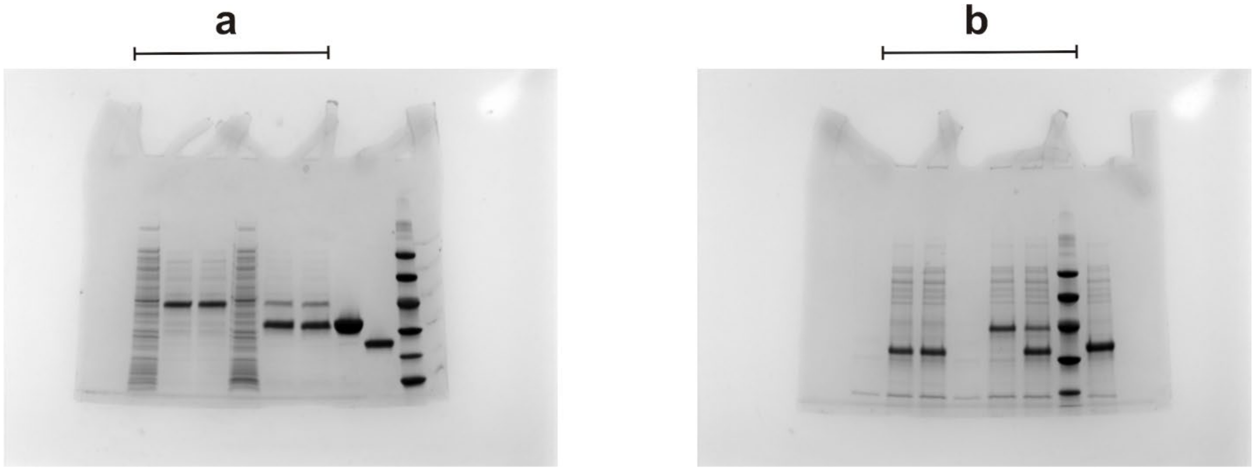


Figure S7. Uncropped gel images of Fig. 2.

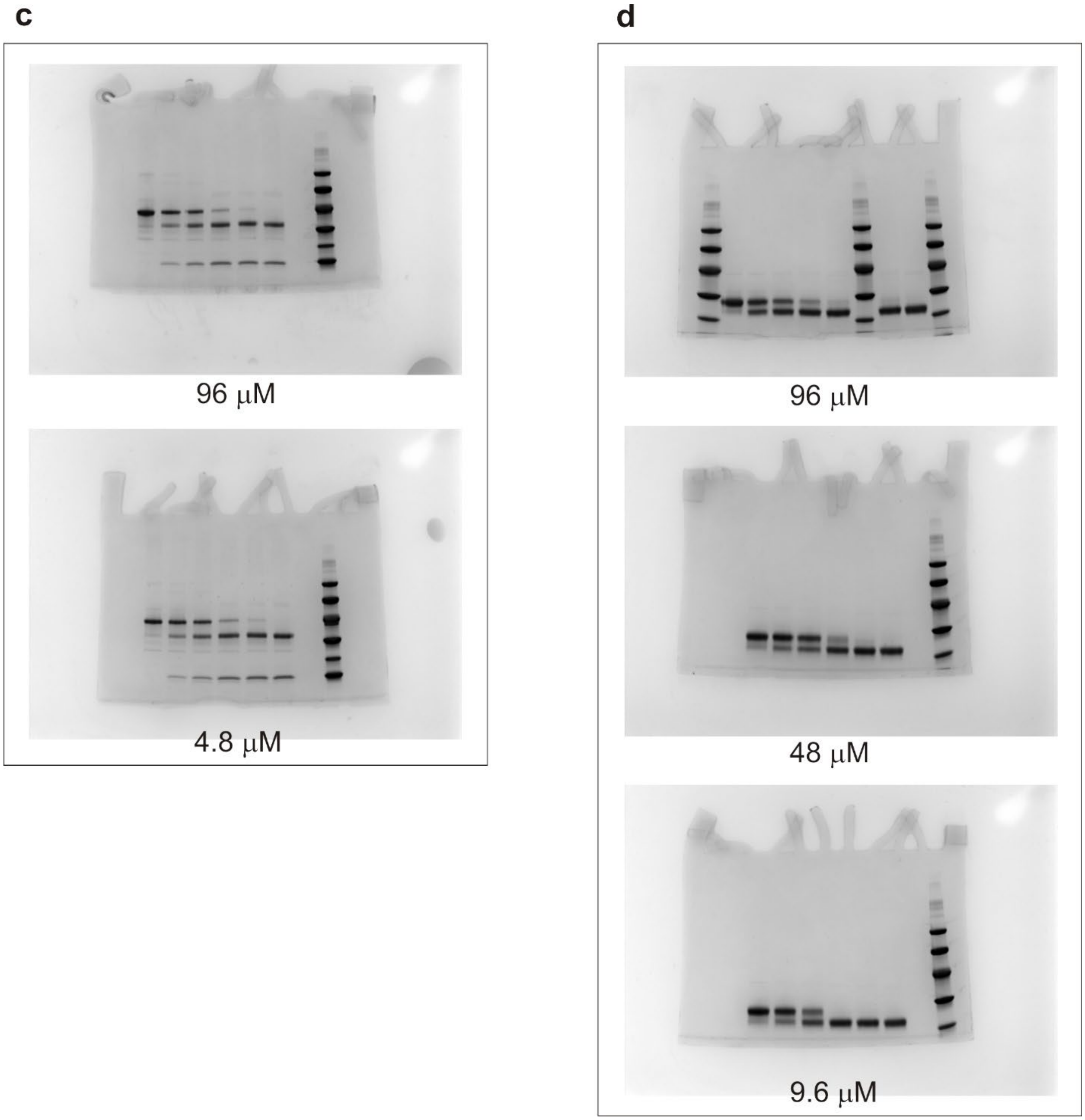


Figure S7. Uncropped gel images of Fig. 3.

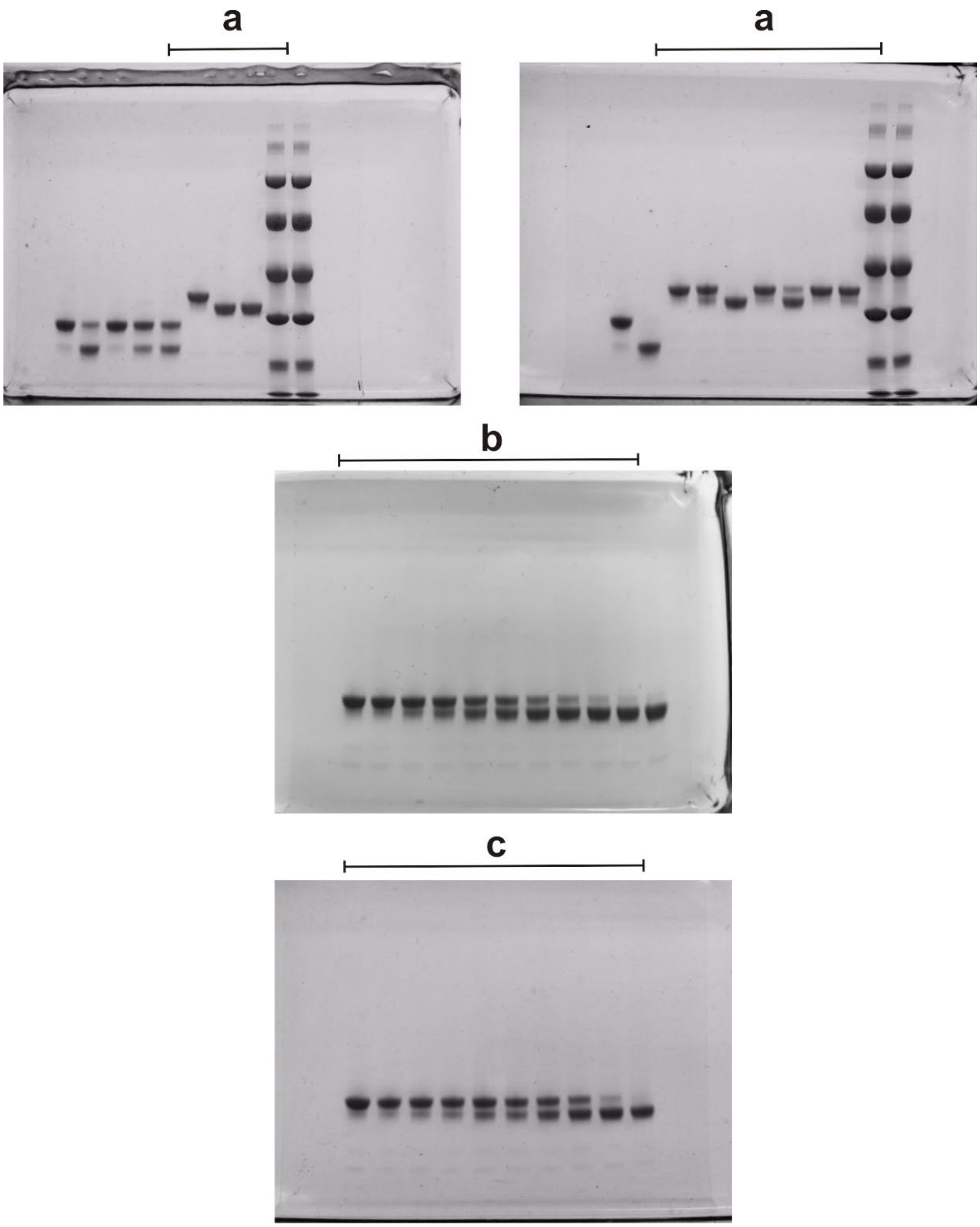


Figure S7. Uncropped gel images of Fig. 4.

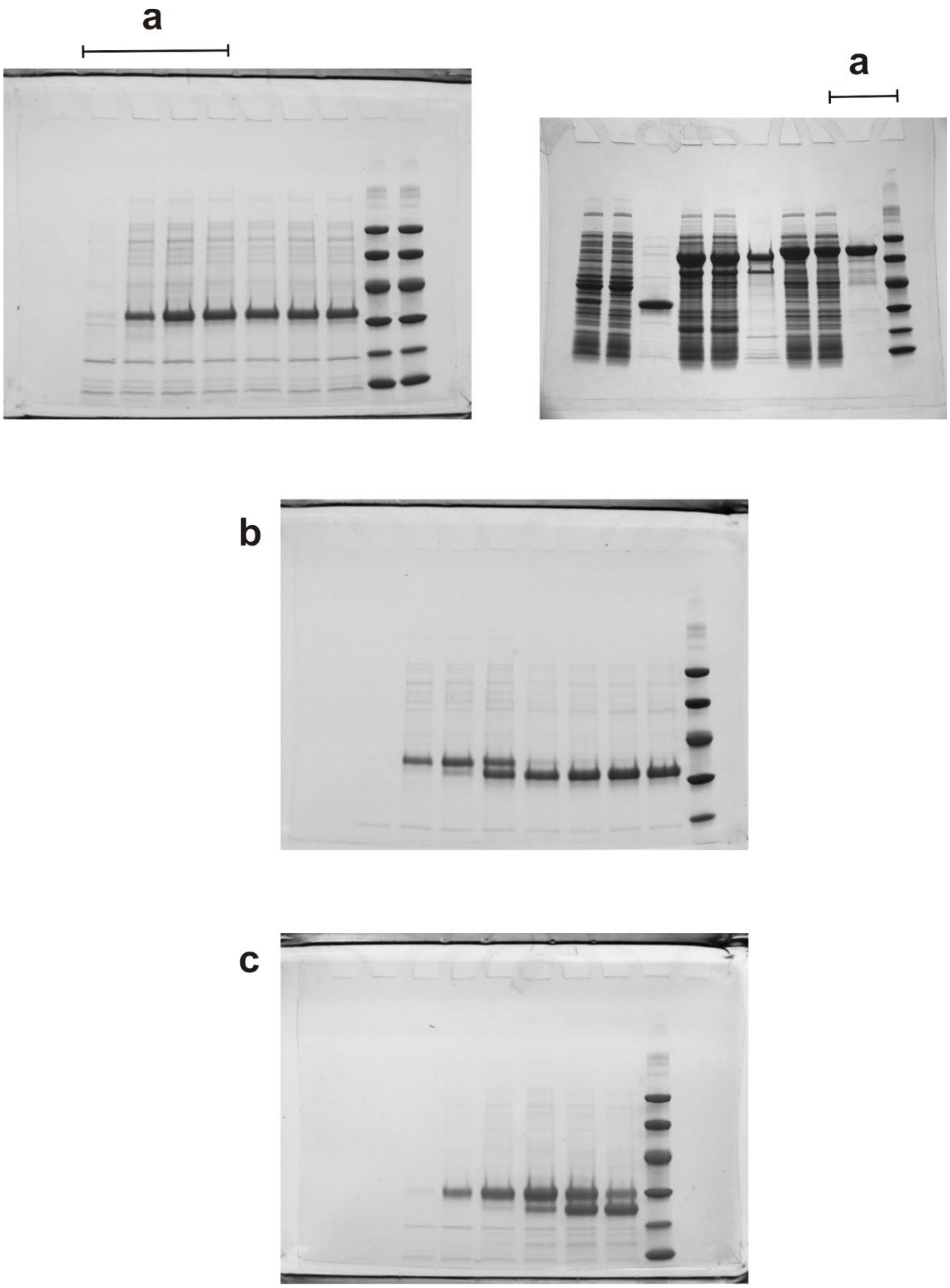


Figure S7. Uncropped gel images of Fig. S2.

**References**

1. Wingfield PT. N-Terminal Methionine Processing. *Curr Protoc Protein Sci* **88**, 6 14 11-16 14 13 (2017).

2. Hirel PH, Schmitter MJ, Dessen P, Fayat G, Blanquet S. Extent of N-terminal methionine excision from Escherichia coli proteins is governed by the side-chain length of the penultimate amino acid. *Proc Natl Acad Sci U S A* **86**, 8247-8251 (1989).

3. Nashed NT*, et al.* Autoprocessing and oxyanion loop reorganization upon GC373 and nirmatrelvir binding of monomeric SARS-CoV-2 main protease catalytic domain. *Commun Biol* **5**, 976 (2022).

4. Kneller DW*, et al.* Unusual zwitterionic catalytic site of SARS-CoV-2 main protease revealed by neutron crystallography. *J Biol Chem* **295**, 17365-17373 (2020).
